# Predicting the Distribution of Mesophotic Coral Ecosystems in the Chagos Archipelago

**DOI:** 10.1002/ece3.71130

**Published:** 2025-04-02

**Authors:** Clara Diaz, Kerry L. Howell, Kyran P. Graves, Adam Bolton, Phil Hosegood, Edward Robinson, Nicola L. Foster

**Affiliations:** ^1^ School of Biological and Marine Sciences University of Plymouth Plymouth UK

**Keywords:** Central Indian Ocean, ecology, independent validation, maximum entropy modelling, mesophotic, species distribution modelling

## Abstract

To support conservation efforts, accurate mapping of marine organism community’ distribution has become more critical than ever before. While previous mapping endeavours have primarily focused on easily accessible shallow‐water habitats, there remains limited knowledge about the ecosystems lying beyond SCUBA diving depths, such as mesophotic coral ecosystems (MCEs, ~30–150 m). MCEs are important habitats from an ecological and conservation perspective, yet little is known about the environmental factors that shape these ecosystems and their distribution, particularly in the Indian Ocean region. The goals of this study are to (1) predict the spatial distribution and extent of distinct benthic communities and MCEs in the Chagos Archipelago, central Indian Ocean, (2) test the effectiveness of a range of environmental and topography derived variables to predict the location of MCEs around Egmont Atoll and the Archipelago, and (3) independently validate the models produced. In addition, we compared the MCEs predicted extent in the Archipelago for the models derived from high‐resolution multibeam and low‐resolution GEBCO bathymetry data. Using maximum entropy modelling, all models resulted in excellent (> 0.9) performances, for AUC and threshold‐dependent metrics, predicting extensive and previously undocumented MCEs across the entire Archipelago with, however, differences in the predicted extent between the high‐ and low‐resolution models. Independent validation resulted in fair (> 0.7 AUC) and poor (> 0.6 AUC) performances for the high‐resolution and low‐resolution models, respectively. Photosynthetically active radiation (PAR), temperature, chlorophyll‐a, and topographically derived variables were identified as the most influential predictors. In conclusion, this study provides the first prediction of the distribution of MCEs and their distinct benthic communities in the Archipelago. It highlights their significance in terms of potential extent and response to various environmental factors, supporting decision making for prioritising future survey sites to study MCEs across the Archipelago and targeting ecologically important areas for conservation.

## Graphical abstract

This study aims to map and predict the distribution of mesophotic coral ecosystems (MCEs) in the Chagos Archipelago, Indian Ocean, using high‐ and low‐resolution bathymetry data. The models, developed using environmental and topographical variables, showed excellent predictive performance, revealing extensive, previously undocumented MCEs; though independent validation showed stronger results for high‐resolution data. The findings offer valuable insights for conservation efforts by highlighting the ecological importance of MCEs and guiding future research and conservation priorities in the region.

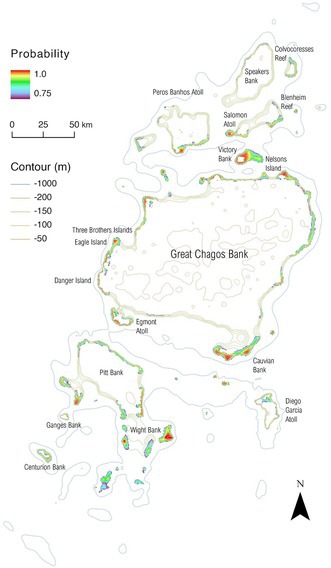

## 1 Introduction

Human‐induced factors (i.e., coastal development, over‐ and destructive‐fishing, land‐based pollution, seabed exploitation, and climate change) have led to great biodiversity losses in the marine realm worldwide (Bellwood et al. 2004), urging scientists to deepen their understanding of underwater habitats. To support conservation efforts, the development of accurate maps depicting the spatial distribution of marine organism communities has become more critical than ever before (Howell et al. 2022). While previous mapping endeavors have primarily focused on easily accessible shallow‐water environments (Andréfouët et al. 2006), there remains limited knowledge about the ecosystems lying beyond SCUBA diving depths compared to shallow‐water habitats (Turner et al. 2017). These deeper habitats present significant challenges, requiring costly and difficult techniques to obtain high‐resolution data over large spatial ranges (Woodall et al. 2018).

Mesophotic Coral Ecosystems (MCEs) typically occur between 30 and 150 m in tropical and subtropical regions and have gained interest in the scientific community in recent decades due to advances in technology making these ecosystems more accessible (Pyle and Copus 2019; Radice et al. 2024). Research has found that MCEs host rich biodiversity (Liddell and Ohlhorst 1988; Stefanoudis et al. 2019; Montgomery et al. 2021; Diaz et al. 2024) play a key role in ecosystem services provision (Holstein et al. 2019) and are distinct from shallow‐water reefs (Lesser et al. 2019; Stefanoudis et al. 2019; Diaz et al. 2024). However, MCEs face threats from climate change (Diaz et al. 2023a) and direct anthropogenic impacts (Holstein et al. 2019), underscoring the urgency to learn more about these important ecosystems. Despite recent research, our understanding of MCEs, such as the environmental factors that shape these ecosystems (Turner et al. 2017) and where MCEs are distributed, remains limited, particularly in the Indian Ocean region compared to other parts of the world (Pyle and Copus 2019). These knowledge gaps have significant implications for marine spatial planning, where MCEs may be overlooked or incidentally included in marine protected areas (MPAs) due to geopolitical boundaries, rather than explicitly due to their ecological significance (Stefanoudis et al. 2022). Evidence‐based marine spatial management is crucial to ensure the most appropriate protection for marine ecosystems. To bridge this knowledge gap, habitat‐suitability modelling (HSM) and species distribution modelling (SDM) offer a promising solution. These models use statistical or machine learning techniques to predict species presence, absence, abundance, or biomass for instance, based on species data and relevant environmental parameters, enabling the mapping of potential species distribution in data‐sparse areas (Silva and MacDonald 2017; Howell et al. 2022). This approach has proven to be particularly valuable in studying organisms that are logistically difficult to sample on a large scale, such as MCEs or deep‐sea habitats (Costa et al. 2015; Ross et al. 2015; Nolan et al. 2024). HSMs are a rapid and cost‐effective tool that can help unravel two research interests: first, a better understanding of which environmental factors are the most important drivers of a species' distribution and second, predicting the distribution of investigated species over a defined area (Guisan and Zimmermann 2000). To date, modelling approaches are the main methods used to map benthic communities in deep water (Howell et al. 2022).

Predictive modelling, while still in its infancy, has proven to be a valuable tool to address knowledge gaps surrounding MCEs (Bridge et al. 2012; Costa et al. 2015; Silva and MacDonald 2017; Sterne et al. 2020; Swanborn et al. 2022). By identifying the key environmental and geomorphological factors influencing MCE occurrence, these models offer insights into their distribution patterns. However, most of the studies aiming to model MCEs focused on specific taxa occurring within these habitats (Bridge et al. 2012; Costa et al. 2015; Silva and MacDonald 2017; Sterne et al. 2020), rather than incorporating multiple species to represent the full spectrum of benthic communities within MCEs (Swanborn et al. 2022).

In this study, we aimed to (1) predict the environmental drivers of MCE occurrence at both the individual benthic community and MCE levels using the maximum entropy (MaxEnt) modelling technique, (2) gain a deeper understanding of the distribution of MCEs in this region by employing two scales of terrain metrics, and (3) independently validate the models produced. Our focus encompassed Egmont Atoll and the wider Chagos Archipelago in the Indian Ocean. To achieve our objectives, we utilised a combination of environmental, bathymetry, and underwater video data.

## 2 Methods

### 2.1 Study Area

**FIGURE 1:**
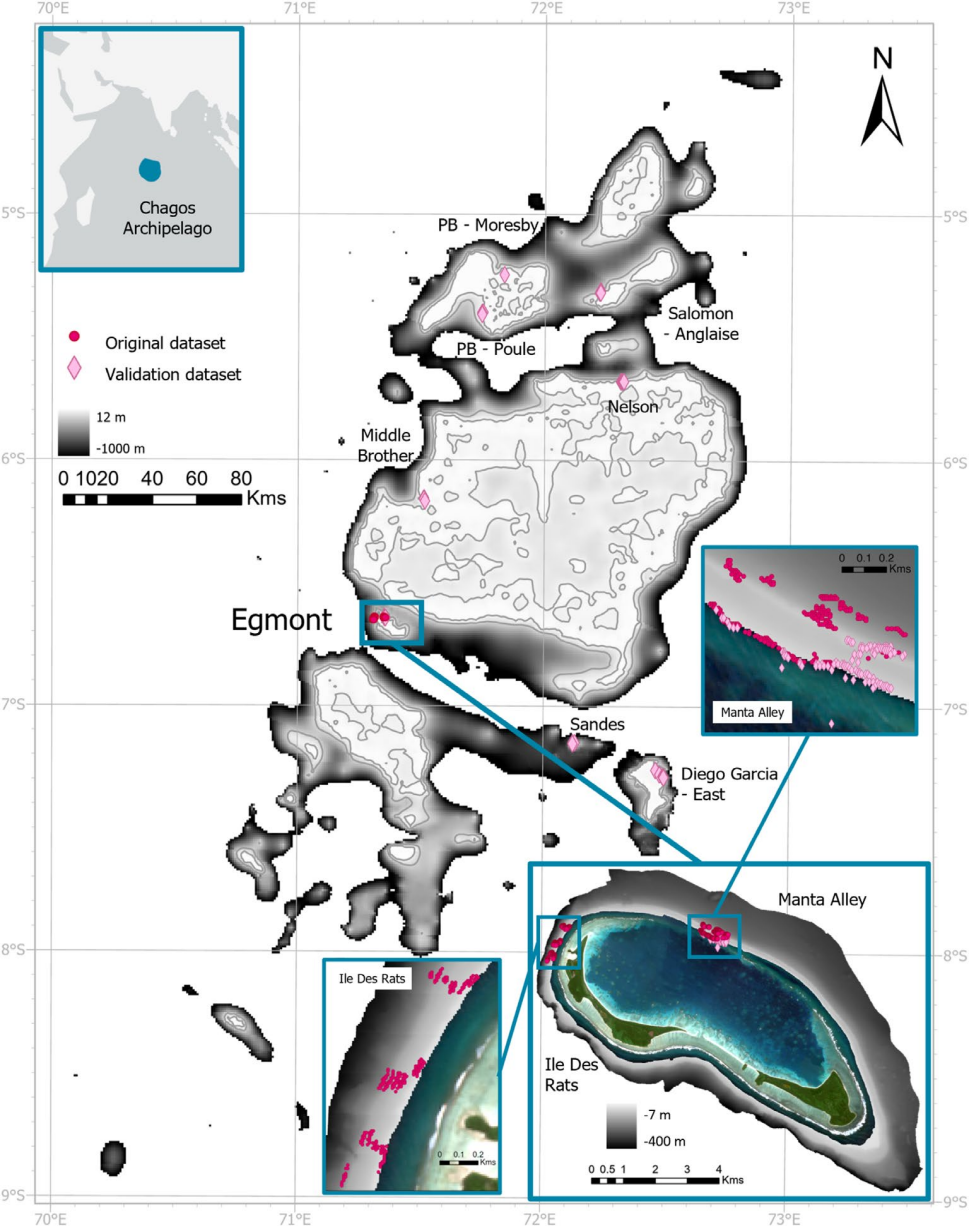
Composite figure of the Chagos Archipelago (main figure) located within the central Indian Ocean (left corner inset) and Egmont Atoll (right corner inset). The figure shows the original data set (dark pink circles) together with the independent validation data set (light pink diamonds) used to build the high resolution (Egmont) and low resolution (all Archipelago) models. The Chagos marine protected area is displayed in teal on the left corner inset. The bathymetry (in meters) was extracted from GEBCO (General Bathymetric Chart of the Oceans; here shown from 12 to −1000 m; 472 m resolution) data for the Archipelago, with −30 and −150 m depth contours displayed in light grey; and acquired using multibeam (from −7 to −400 m; 9 m resolution) for Egmont Atoll. Manta Alley and Ile Des Rats were the sites used for the original data set (see the insets detailing the sampling areas for each site); and Diego Garcia—East; Sandes Seamount; Nelson Island; Salomon Atoll—Ile Anglaise; PB (Peros Banhos)—Ile Moresby; PB—Ile Poule; Middle Brother Island and Manta Alley were the sites used for the validation data sets.

The Chagos Archipelago (6° S, 71°30′ E) is considered a hotspot of marine life in the middle of the Indian Ocean due to both its remote location and the creation of a fully no‐take MPA in 2010, resulting in minimal direct human influence (Head et al. 2019; Hays et al. 2020). This study focused on Egmont Atoll (6°40′ S, 71°21′ E) (Figure 1) and the wider Archipelago. Two sites were surveyed at Egmont Atoll: Ile Des Rats (IDR), located on the North‐West coast, and Manta Alley (MA), located on the North‐East coast (Figure 1). These two sites were selected based on their contrasting oceanographic regimes, with IDR open to the wider ocean, while MA is somewhat sheltered from the Great Chagos Bank. Both sites occur on steep slopes with rocky outcrops and strong currents prevailing from east to west and west to east, depending on the movement of the tide. Biological and multibeam data were collected at these two sites during late November 2019 and late March 2020.

### 2.2 Biological Data Collection and Preparation

A Saab Seaeye Falcon Remotely Operated Vehicle (ROV) was used to collect benthic imagery of MCEs in the Archipelago. It was equipped with four SAAB Seaeye LED daylight white lamps (34 W, 3520 Lumens) and two cameras: one recording live video in standard resolution (720 p; functioning with low light and a wide angled lens), with an information overlay displaying time, depth, heading, pitch, and roll; and a second one (GoPro hero 4), positioned directly under the SAAB Seaeye camera and recording footage in high definition (2.7 k, 24 fps, wide field of view). Dive duration was limited by the GoPro battery length (~ 3 h).

Still images were sampled parallel to the slope for practical purposes and due to the presence of a strong current around the Atoll, rendering benthic linear video transects difficult to undertake. This method was equivalent to a belt transect approach where image “quadrats” were sampled at intervals along the transect. To collect image samples, the ROV descended to the appropriate depth zone and approached the seabed with the cameras positioned at an oblique angle (altitude < 1.5 m). Still frames were captured to collect high‐quality images allowing benthic specimen identification and maximising the field of view of the seabed. For each image, time, depth, and sampling position (via a mounted acoustic ultra‐short baseline (USBL) transponder system) were recorded. In this study, image extraction intervals within a ‘transect’ and between depth bands were not identical, but images were instead selected based on their suitable position and quality; further information on each image and ‘transects’ can be found in the raw materials provided in the ‘data availability’ section.

In this study, six depth bands were surveyed, chosen a priori to the study and based on descriptions of community characteristics from the literature, covering the shallow reef down to the lower mesophotic zones: 15–20; 30–40; 60–70; 80–90; 110–120; and 150–160 m. Three ‘transects’ (each consisting of 30 images) were surveyed per depth band, parallel to the slope at each of the two study sites (36 ‘transects’ in total). Ninety images were collected per depth band and per site (1080 images in total). This study focuses on benthic organism counts rather than an area‐based survey.

As the ROV was not equipped with functional paired lasers during the surveys, calculation of the field of view within each image could not be undertaken. To standardise data collection, however, the pilots positioned the ROV at a similar altitude, with the same angle of view, for each collected image. The mean time taken to complete a single ‘transect’ was 30 min 3 s (± 6 min 58 s, with 19–31 min for the 15–20 m transect; 22–41 min for 30‐40 m; 31–38 min for 60–70 m; 19–42 min for 80–90 m; 27–50 min for 110–120 m; and 21–43 min for 150–160 m) which was determined when at least 30 suitable images were collected, with no significant differences observed in the time taken to complete a ‘transect’ with depth and site (Kruskal‐Wallis test, −*p*‐value > 0.05. ‘Transect’ by depth: chi‐squared = 10.244, df = 5, *p*‐value = 0.069; transect by site: chi‐squared = 0.002, df = 1, *p*‐value = 0.962). In addition, data were standardised by individuals and sampling coverage per depth band, as suggested by Colwell et al. (2012) and Roswell et al. (2021), using the iNext package in R (Hsieh et al. 2020).

Still images were annotated using BIIGLE 2.0, an online annotation platform (Langenkämper et al. 2017), which enables identification and enumeration of organisms. In this study, all benthic organisms (≥ 1 cm) within each image were identified and quantified, with encrusting algae estimated as percentage cover on the image. As the standard taxonomic approaches are not often applicable to image data, all organisms were identified to the highest taxonomic resolution possible, and distinct morphospecies were assigned an operational taxonomic unit (OTU), following the method in Howell et al. (2010). A morphospecies may hence correspond to species, genus, family, or higher taxonomic levels. Alongside the image analysis, a morphospecies catalogue was created for the region (Diaz et al. 2023b) following the global standardised marine taxon reference image database suggested by Howell et al. (2019), with a total of 582 OTUs identified. Quality assessment of the image annotation was undertaken by the same observer, who annotated 5% of the total number of images, as suggested by Schoening et al. (2016). Data from the two annotation sessions were assessed for accuracy and reproducibility of annotations: images were compared two by two (original and re‐annotated images) with a similarity of percentage (SIMPER) analysis and visualised with a non‐metric multidimensional scaling (nMDS) using PRIMER v.6 (based on square root transformed data and Bray–Curtis similarity). The SIMPER analysis revealed a similarity of 76.9%–90.1% between the two images within transects, which is considered an acceptable consistency in the annotation process. Furthermore, annotations were verified by a second observer (> 50%).

Hierarchical clustering with a similarity profile (SIMPROF) permutational test based on square‐root transformed data, with Bray–Curtis similarity, was conducted at the transect level to identify clusters of statistically significant distinct benthic assemblages using the statistical software package PRIMER v.6 (Clarke and Gorley 2006) with PERMANOVA+ add‐in (Anderson et al. 2008). The resulting plot can be visualised in Supporting Information S1. Clusters falling within 55% of similarity were grouped together (Supporting Information S1) as a trade‐off between the minimum data point number required to undertake species distribution modelling analyses and ecologically relevant groupings based on previous studies. Hence, IDR and MA were grouped for the same depth band, and one transect from 150 to 160 m deep (MA, transect 3) was excluded from the analysis. In addition, a single cluster of the benthic communities residing in the mesophotic zone was created based on the MCE traditional upper and lower boundary definition (30–150 m), by grouping the communities within the 30–40 m, 60–70 m, 80–90 m, 110–120 m, and 150–160 m transects from the two study sites. This cluster was created to predict the distribution of MCEs across the Archipelago.

### 2.3 Environmental Predictors

High‐resolution bathymetry data were obtained from a R2Sonic multibeam echosounder at a 9 × 9 m resolution, using QPS QINSy (9.2) and cleaned manually with QPS Qimera (2.1) for Egmont Atoll (https://qps.nl/qinsy/) (depth range 7–400 m, Figure 1). The data were gridded using the “nearest neighbour” algorithm. Low‐resolution bathymetry data were obtained from the GEBCO (General Bathymetric Chart of the Oceans) 2023 15 arc‐s grid (472 × 472 m) for the entire Chagos Archipelago. The bathymetry data extracted from GEBCO must be taken with caution, as it is mostly satellite‐derived, with extrapolations made from the few sampled places in the Indian Ocean (please see Section 4.3 ‘Methodological Considerations’ section). In particular, bottom slopes are much shallower in GEBCO interpolation compared to reality (i.e., as demonstrated by the multibeam surveys undertaken in Egmont Atoll, Figure S22). In addition, scaling issues can arise when using low‐resolution bathymetry data. To address this, GEBCO data were only used for the MCE cluster, grouping all the transects as well as a large depth range (30–150 m), spanning a larger area than a single GEBCO grid cell (Figure 1), but considering that one cell may contain both presence and absence points in some cases. Four topographic variables were derived from both bathymetry layers: broad‐scale bathymetric position index (BBPI), fine‐scale bathymetric position index (FBPI), slope, and ruggedness were created using the Benthic Terrain Modeler extension (Walbridge et al. 2018) in ArcGIS Pro 2.9.5. BBPI was calculated using an inner radius of 5 cells and an outer radius of 250 cells, resulting in a scale factor of 2.2 km using high‐resolution data and 115.6 km using low‐resolution data. FBPI was calculated with an inner radius of 3 cells and an outer radius of 25 cells, resulting in a scale factor of 0.2 km using high‐resolution data and 10.4 km using low‐resolution data. Slope and ruggedness (also called rugosity) were calculated with a neighbourhood size of 3. BPI gives the relative elevation of a point in relation to the overall landscape (Lundblad et al. 2006): positive values indicate features rising above the surrounding terrain, such as ridges and pinnacles, while negative values indicate depressions, such as gullies and canyons. In contrast, areas with constant slope or flat areas are represented by near‐zero values. BPI acts as a surrogate for various environmental factors that affect species distribution, such as light or current exposure, current speed, and sedimentation, without the confounding effects of other variables (e.g., temperature and salinity) (Evans et al. 2015).

Temperature, salinity, and chlorophyll‐a (Chl‐a) were extracted from publicly available data measured with Argo floats (https://argo.ucsd.edu/). The Argo floats were selected based on the combination of the data measured, their location (as close as possible to the Archipelago), and the duration of data collection (for at least four years and recently, from January 2018 and January 2022). Hence, data from Argo buoys #1902332, #2902242, #2902179, #2902239, and #2902244 were extracted from 0 to 200 m deep, for all sites. Average temperature, salinity, and Chl‐a were calculated per depth for every Argo float over the four years. In addition, average minimum, maximum, and variability (maximum—minimum, also called delta) temperature data per depth were extracted as described above. Vertical profiles of photosynthetically active radiation (PAR, also called irradiance and calculated in μmol photons m^2^ s^1^, between 400 and 700 nm) were conducted using a LI‐COR LI‐193 spherical underwater quantum sensor. The sensor was attached to a metal lowering frame along with a Star‐Oddi Starmon Temperature/Depth recorder and lowered over the side of the vessel to a maximum depth of 180 m. Nine midday (12:10–13:15) vertical deployments were taken in the Archipelago from the 12th to the 17th of March 2020 and from the 19th to the 29th of March 2022 around Egmont Atoll and Sandes Seamount (7°14′ S,72°14′ E, 95 km East of Egmont Atoll), with a total of 18 profiles recorded (upward and downward profiles of the 9 PAR deployments). Average PAR was calculated per depth for every deployment. Temperature, salinity, Chl‐a, and PAR were then modelled with generalised additive models (GAMs) using the mgcv package in R (Wood 2011) with depth, latitude, and longitude used as explanatory variables. All the environmental data were re‐projected from their original projection (WGS 1984) into Goode Homolosine Ocean (GHO) equal‐area projection, to allow for correct calculation of derived topographic variables and area. Collinearity between environmental variables was tested using Pearson's correlation coefficients using the “cor()” function in the core “stats” package in R, v4.1.0 (R Core Team 2020). Highly correlated variables (> |0.9|) were not used when constructing multivariable models, with the variable allowing for a more intuitive ecological interpretation retained (the correlation matrices are provided in Tables S1 and S2).

### 2.4 Maximum Entropy (MaxEnt) Modelling

Habitat suitability models were constructed using Maximum Entropy (MaxEnt), a machine learning software. For each cluster of distinct benthic communities (1–6, only predicted with the high‐resolution bathymetry data), the dataset was reduced to one point per cell of environmental data in ArcGIS (Figure 1), where each point's value was either 0 (absence) or 1 (presence) for each grid cell. In this study, as the ‘absence’ points were used from the other cluster points only, it is not certain that they are not present in any other cells within a 9 or 472 m grid cell, and therefore, the data within this study represent ‘pseudo‐absences’, instead of ‘true absences’ (Table S3), as used in Howell et al. (2022). In addition, as biological data from clusters were merged to create the overall MCE cluster, it resulted in a high imbalance between the number of presence points and pseudo‐absence points (381 and 39, respectively, for the high‐resolution bathymetry; 12 and 1, respectively, for low‐resolution bathymetry) (Table S3). The reduction in the number of data points was undertaken on ArcGIS, where the initial high‐resolution presence/absence data points' shapefile was added to the low‐resolution bathymetry raster. A raster with the data points was then created by using the “cell assignment type” function set to “maximum” (i.e., a cell was set as a presence cell if > 50% of the points in the cell were presence points). The newly created raster was finally converted to a shapefile, with low‐resolution presence/absence cell and their new coordinate attributes automatically assigned by ArcGIS. Here, a presence‐only modelling approach was adopted for the MCE cluster, a method that has been successfully used in previous studies (Bridge et al. 2012; Ross et al. 2015; Silva and MacDonald 2017; Howell et al. 2022). 10,000 background points, automatically chosen by MaxEnt, were used to represent locations with an equal likelihood of having been sampled, that act as absence points to inform the model (Elith et al. 2011). For each benthic cluster (i.e., distinct benthic communities or overall MCE cluster), the corresponding values of the predictor variables were extracted for each presence, pseudo‐absence, or background cell.

Numerous methods can be used to construct HSMs, including commonly used regression‐based approaches, for example, generalised linear models (GLMs) or generalised additive models (GAMs), and tree‐based approaches, for example, random forest (RF). While there is no “silver bullet” method (Qiao et al. 2015) and ensemble modelling should also be considered, model input data can place limitations on what modelling frameworks can be implemented. In this study, MaxEnt (Phillips et al. 2006, 2017) was selected as the modelling framework because of its ability to utilise small sample sizes (Hernandez et al. 2006; Pearson et al. 2007) and its design to handle presence‐only datasets. Additionally, MaxEnt is often ranked among the highest performing when tested against all other commonly used methods, including GAMs, GLMs, and RF (Valavi et al. 2022).

Variable selection using MaxEnt in R with the “dismo” package (Hijmans and Elith 2023) consisted of backward step‐wise selection, dropping variables one by one from the full model, in the order of the lowest model gain when used in isolation, to the highest, based on the jackknife plots and area under the curve (AUC) results, until the most parsimonious model was found. The selected variables for each cluster can be consulted in Table 2. For each cluster, preliminary models with different regularisation parameters were systematically trialled with the MaxEnt Java software interface (version 3.4.3) to avoid over‐fitting/smoothing (Phillips and Dudík 2008). Jackknife plots, response curves, and AUC details are provided in Table S4 and Supporting Information S3. The final MaxEnt models were projected onto the study area and constrained to sampled conditions using the MaxEnt novel environmental variable output (i.e., areas where the values fall within those on which the model was trained).

### 2.5 Model Evaluation

#### 2.5.1 Model Dataset Partitions

For each individual benthic community cluster, the full high‐resolution model dataset was manually split into training (70%) and test (30%) datasets, a process that was repeated to build 10 new partitioned datasets, which preserved the prevalence between the presence and pseudo‐absence points. A new model was built with each new partition, using the MaxEnt Java program. For both MCE clusters (high‐resolution and low‐resolution), the training and test datasets were obtained using the cross‐validate function on the MaxEnt java program, with the default setting of 10,000 background points selection. The models were replicated 10 and 6 times for the high‐resolution and low‐resolution MCE models, respectively (the number of repetitions was constrained by the number of presence points for the low‐resolution MCE model).

#### 2.5.2 Model Performance Assessment

Each model was assessed using the “PresenceAbsence” package in R (Freeman and Moisen 2008), employing both threshold‐independent (AUC) and threshold‐dependent metrics. Three thresholding techniques were used to assess model performance, according to Liu et al. (2005). Sensitivity‐specificity equality (Sens = Spec), sensitivity‐specificity sum maximisation (MaxSens+Spec), and minimum distance to the left corner in the ROC curve plot (minROCdist) were used in this study. Model performances with each different thresholding method applied were assessed using three indices: sensitivity (Sens.), specificity (Spec.), and percent correctly classified (PCC). Sensitivity relates to the proportion of the presence observations predicted correctly as presence, while specificity relates to the proportion of the absence observations that were correctly predicted as absences. PCC is the number of correctly classified observations (presence and absence) as a percentage of the total number of observations. Considering the averaged threshold‐dependent metrics for the partitions together with full model metrics, a final threshold was chosen to maximise final model performance. The best model performance was determined by the highest average score across all measured indices, as well as following the recommendations of Liu et al. (2016). Values were classified on a five‐point scale: excellent (1–0.9), good (0.9–0.8), fair (0.8–0.7), poor (0.7–0.6), and fail (0.6–0.5) (Ross et al. 2015). For both AUC and threshold‐dependent metrics, the mean and standard deviation (SD) were calculated for partitioned datasets and the full models.

The chosen thresholding method was selected for use in the final models. In addition to the probability of predicted presence maps, a binary map of predicted presence and pseudo‐absence, based on the threshold value selected, was produced for each model. Relative likelihoods that fell below the selected threshold for each individual benthic cluster and the MCE cluster (Table S1) were converted to an absence raster (cell value of 0), while relative likelihoods falling above the threshold were converted to a presence raster (cell value of 1). In addition, SD maps were produced from the relative probability maps from all the partitioned test/training models to display spatial uncertainty in the model predictions (Supporting Information S5).

Spatial autocorrelation was tested using the average nearest neighbour function in ArcGIS Pro to provide an indication of the degree of spatial dispersion in the distribution, based on the Euclidean distance between points.

#### 2.5.3 Quantification of MCE Community Distribution

The number of predicted presence cells within each binary map for Egmont Atoll and the Chagos Archipelago was calculated and then expressed as surface area. Finally, the surface extent of grouped distinct benthic communities and the entire MCE community based on high‐resolution multibeam data was estimated for the entire Archipelago. This was done by comparing the difference in the predicted surface extent of communities between high‐resolution models and low‐resolution models at Egmont Atoll only and then multiplying the predicted surface extent of MCE communities from low‐resolution models for the entire Archipelago by this difference factor. As the models created here are based on environmental similarities, we assumed that the same factor holds across different areas with similar environmental factors.

### 2.6 Independent Model Validation

Additional biological data were collected around the Archipelago in December 2019 (at Sandes Seamount, between 60 and 90 m depth) using the Falcon SeaEye ROV, as described in the previous section and in January–February 2024 (Egmont Atoll—Manta Alley; Nelson Island; Peros Banhos—Ile Moresby and Ile Poule; Middle Brother Island; Diego Garcia Atoll‐East; Salomon Atoll—Ile Anglaise) (Figure 1), using a Drop Camera system. These additional observations were not conducted for the sole purpose of model validation, but this was a consideration during data collection. The Drop Camera system consisted of a metal frame equipped with a GoPro hero 7 in a deep housing facing downward, a Nanight torch, and a Star Oddi TD (temperature‐depth) sensor. The Drop Camera system was lowered over the side of the vessel in a predetermined survey location. Time‐lapse photography was used to collect images of the seabed every 0.5 s, with clear frames close to the seabed extracted at 20, 40, 60, and 90 m depth along the slope on the outer reefs. The breakdown in the data point number per site for the model construction and model validation can be consulted in Table S3.

The new biological data sets for validation were plotted in ArcGIS Pro on raster grids of the two original MCE models (high and low resolution) in GHO to match this study's projection. The validation datasets were reduced to one point per cell (Figure 1), where each point's value was either 0 (absence) or 1 (presence) for each grid cell. As the original models were masked for novel climates, validation data points that were outside of the model predictions were removed, as they were considered outside of the original model domain. A total of 71 and 27 validation points were used for the MCE high‐resolution and low‐resolution models, respectively (Table S3). The probability values from the two models were extracted for each validation data point. Threshold‐dependent and threshold‐independent metrics were calculated and compared to the original models, using both the threshold defined in the original models and a new threshold that maximised model performance against the new dataset.

## 3 Results

### 3.1 Cluster Composition

**FIGURE 2:**
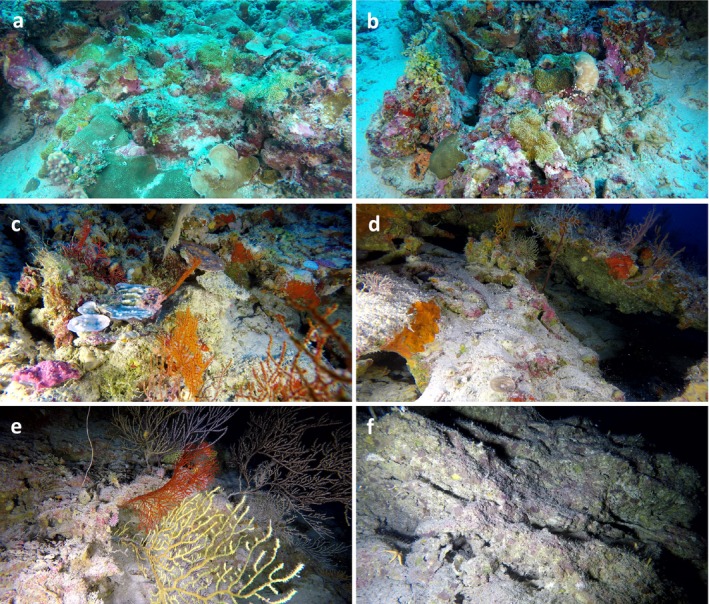
Representative benthic community for each cluster: (a) Cluster 1 (15–20 m); (b) Cluster 2 (30–40 m); (c) Cluster 3 (60–70 m); (d) Cluster 4 (80–90 m); (e) Cluster 5 (110–120 m); and (f) Cluster 6 (150–160 m), at Egmont Atoll.

Six clusters of distinct benthic communities at Egmont Atoll were identified and selected with a SIMPROF test, at 55% similarity (Supporting Information S1; clusters 1–6; Table 1; Figure 2). A SIMPER analysis performed on these clusters determined the taxa responsible for both discriminating and characterising these communities (Table 1). Cluster 1 was dominated by zooxanthellate scleractinian corals and calcareous macroalgae from the genus *Halimeda* (Table 1; Figure 2a). Cluster 2 was dominated by sponges and the same calcareous macroalgae, with numerous zooxanthellate scleractinian corals too (Table 1; Figure 2b). Cluster 3 contained sponges, zooxanthellate scleractinian corals, mostly from the *Leptoseris* genus, as well as octocorals (Table 1; Figure 2c). Cluster 4 contained sponges, octocorals, and hard corals from the *Leptoseris* genus exclusively (Table 1; Figure 2d). Cluster 5 was dominated by octocorals from the *Nicella* genus and hydrozoans from the *Stylasteridae* family (Table 1; Figure 2e). Finally, cluster 6 consisted mainly of encrusting sponges and algae (Table 1; Figure 2f). A detailed assemblage composition, as well as the number of morphospecies and individuals per taxon along the depth gradient, can be found in Diaz et al. (2023c) and Diaz et al. (2024). However, it is worth noting that the taxonomic identifications are coarse, as they were identified with image material only, and may include several species within a morphospecies.

**TABLE 1:** Taxa associated with each cluster, contributing to a minimum 3% of within‐cluster similarity of the SIMPER analysis.

Species	Average abundance	Average similarity	Similarity SD	Contribution	Cumulated contribution
Cluster 1 (15–20 m), average similarity within cluster: 66.51%					
OTU 97_*Pavona* sp.	13.15	4.18	11.08	6.28	6.28
OTU 379_*Porites* sp.	9.16	2.72	9.36	4.09	10.37
OTU 58_Corallinales	7.88	2.65	12	3.98	14.34
OTU 114_Plantae	6.53	2.19	17.99	3.3	17.64
OTU 308_*Halimeda* sp.	7.79	2.09	2.84	3.15	20.79
OTU 44_*Leptoseris* sp.	6.79	2.09	8.96	3.14	23.93
OTU 309_Porifera	6.97	2.08	9.88	3.12	27.05
Cluster 2 (30–40 m), average similarity within cluster: 61.66%					
OTU57_Porifera	9.1	3.81	8.02	6.17	6.17
OTU309_Porifera	8.63	3.75	9.67	6.08	12.25
OTU58_Corallinales	8.1	3.57	17.96	5.79	18.04
OTU114_Plantae	7.68	3.39	8	5.5	23.54
OTU229_Porifera	6.77	2.76	4.77	4.47	28.02
OTU192_Porifera	6.08	2.55	11.35	4.14	32.16
OTU308_*Halimeda* sp.	5.45	2.02	4.96	3.28	35.44
Cluster 3 (60–70 m), average similarity within cluster: 59.20%					
OTU192_Porifera	14.84	5.19	8.8	8.76	8.76
OTU64_Porifera	12	4.07	2.64	6.88	15.64
OTU69_Porifera	10.17	3.88	9.05	6.56	22.19
OTU114_Plantae	9.19	3.48	16.39	5.88	28.07
OTU57_Porifera	9.56	3.36	8.91	5.68	33.75
OTU58_Corallinales	6.39	2.59	11.06	4.38	38.13
OTU259_Porifera	6.93	2.58	14.27	4.37	42.5
OTU56_Porifera	7.45	2.38	2.53	4.02	46.52
OTU340_*Leptoseris* sp.	7.25	2.18	2.59	3.69	50.21
Cluster 4 (80–90 m), average similarity within cluster: 59.90%					
OTU 6_Porifera	14.11	6.21	16.48	10.37	10.37
OTU57_Porifera	12.55	5.64	16.46	9.41	19.78
OTU192_Porifera	13.34	5.4	5.71	9.01	28.79
OTU114_Plantae	8.83	3.91	10.83	6.52	35.31
OTU 4_Porifera	9.48	3.38	3.31	5.64	40.95
OTU340_*Leptoseris* sp.	8.88	2.94	1.59	4.9	45.85
OTU 8_Corallinales	6.44	2.87	9.23	4.8	50.64
Cluster 5 (110–120 m), average similarity within cluster: 62.68%					
OTU17_*Nicella* sp.	9.72	5.19	3.32	8.28	8.28
OTU19_Stylasteridae	10.35	5.12	2.74	8.17	16.45
OTU114_Plantae	8.29	5.05	19.4	8.05	24.5
OTU56_Porifera	7.92	4.46	10.52	7.12	31.61
OTU58_Corallinales	6.84	4.08	9.03	6.51	38.12
OTU64_Porifera	7.05	3.65	5.39	5.83	43.95
OTU57_Porifera	5.98	3.18	4.41	5.08	49.03
OTU162_Alcyonacea	5.17	2.65	3.65	4.23	53.25
Cluster 6 (150–160 m), average similarity within cluster: 62.02%					
OTU114_Plantae	7.06	8.56	8.76	13.8	13.8
OTU59_Porifera	8.08	8.27	5.38	13.34	27.14
OTU64_Porifera	5.62	6.75	5.68	10.89	38.02
OTU58_Corallinales	5.5	6.52	4.23	10.51	48.54
OTU122_Porifera	4.92	5.34	5.56	8.61	57.14

*Note:* Similarity SD: Similarity standard deviation; average abundance, average similarity, contribution, and cumulated contribution are in percentage. OTU denotes operational taxonomic unit. Average similarity is expressed in percentage. An example image of each taxon can be found in Diaz, et al. (2023b).

### 3.2 Predictor Contributions to Model Distributions

Overall, the main determinants of cluster occurrence, for both individual benthic communities and MCEs at Egmont Atoll and the whole Archipelago, were light (PAR) and temperature (either average temperature, temperature variability or both) and their correlated variables, depth, BBPI, and salinity (Table 2, Supporting Information S2). In addition, Chl‐a played a role in the distribution of benthic communities for clusters found between 60 m and 160 m, as well as for MCEs predicted on high‐resolution bathymetry (Table 2). Cluster 6 and MCE high‐resolution distributions were additionally influenced by maximum temperature (Table 2). Light, mean temperature, and Chl‐a decreased with clusters from increasing depth, while temperature variability increased with clusters of increasing depth (Supporting Information S3). Topography‐derived variables (i.e., geomorphology) were not included in the most parsimonious models for individual significant clusters (clusters 1–6) but played a role for the two MCE models. Indeed, FBPI, BBPI, and slope were found to play a dominant role in the distribution of MCEs, at both high‐resolution (for FBPI and slope) and low‐resolution bathymetry data (for FBPI and BBPI) (Table 2). For all models, the order of variable importance in terms of model gain is provided in Supporting Information S3, depicted as jackknife plots as well as response curves of individual variables for each model.

**TABLE 2:** Model variable selection.

Cluster	Variables selected	Regularisation multiplier
1	PAR, T mean, T delta	1
2	PAR, T mean, T delta	1
3	PAR, T mean, Chl‐a	1
4	PAR, T mean, T delta, Chl‐a	1
5	PAR, T mean, T delta, Chl‐a	1
6	T mean, T max, Chl‐a	3
MCEs (high)	PAR, T mean, T delta, T max, Chl‐a, FBPI, slope	1
MCEs (low)	PAR, T delta, FBPI, BBPI	0.5

*Note:* Chl‐a: chlorophyll‐*a*; PAR: photosynthetically active radiation; T mean, max, delta: temperature average, maximum, variability (max–min); BBPI and FBPI: broad & fine‐scale benthic position index. Regularisation multiplier: Degree of smoothing on each of the variables, to avoid overfitting or overgeneralising the response curves of each variable.

**FIGURE 3:**
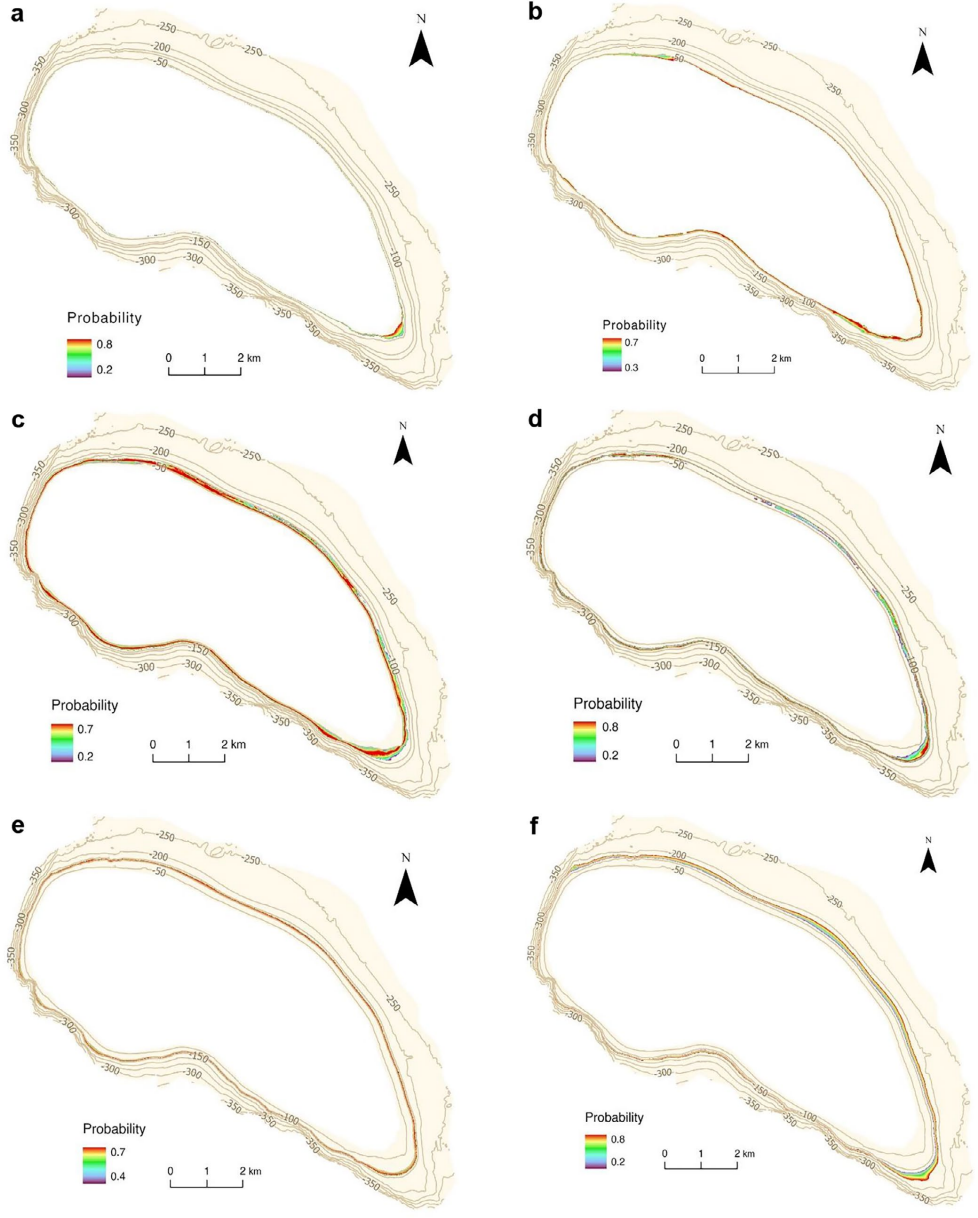
Spatial predictions for each cluster assemblage at Egmont Atoll using the MaxEnt modelling technique. With depth contours at 50 m intervals, starting at 50 m deep. (a) Cluster 1; (b) Cluster 2; (c) Cluster 3; (d) Cluster 4; (e) Cluster 5; and (f) Cluster 6. Predicted standard deviation maps are available in Figure C.18. Beige background indicates predicted absence. White background indicates the Egmont Atoll shape.

Individual community cluster maps overall followed the depth contour around Egmont Atoll, as shown in the prediction and presence maps with thresholding selected probabilities (Figures 3 and S2, respectively), as the main environmental drivers were highly correlated to depth (Supporting Information S2) and the clusters were cut off at the depth band level. While probability scales differed among the predictability maps, they consistently displayed 70% to 80% probability at their highest (Figure 3). Clusters 1 (15–20 m) and 2 (30–40 m) were the least visible on the map, as they were located at the edge of the multibeam area surveyed (Figure 3a,b). In addition, high probabilities of presence in areas with gentle slopes were observed (depicted by larger distances between depth contour lines), with the southeast tip of Egmont predicted to contain every cluster, with a relatively large spatial area for clusters 1; 3 (60–70 m); 4 (80–90 m); and 6 (150–160 m) (Figure 3a,c,d,f).

**FIGURE 4:**
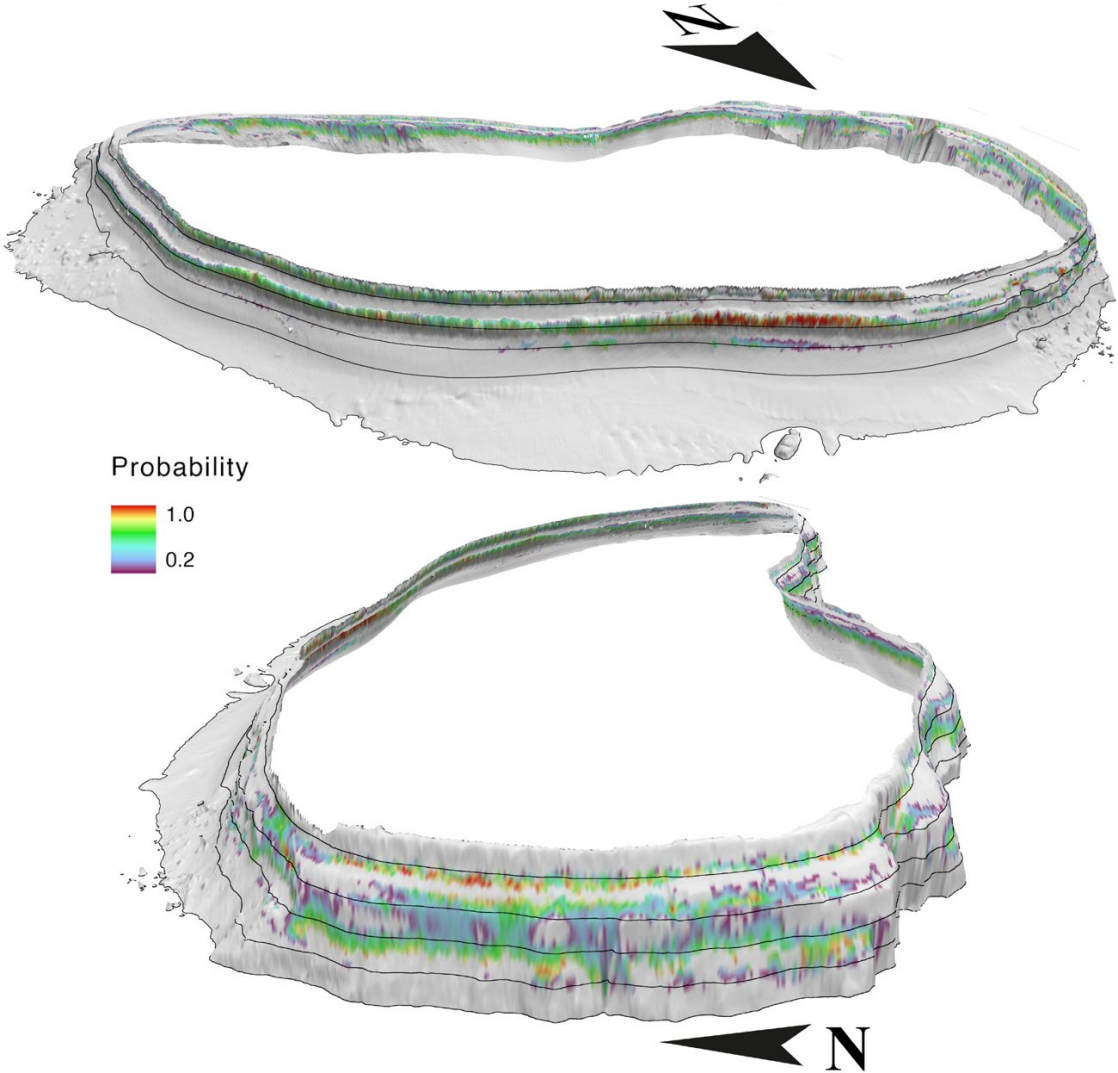
Spatial predictions for MCE (30–160 m) – high‐resolution 3D bathymetry cluster at Egmont Atoll using MaxEnt modelling technique, seen from Manta Alley (top) and Ile Des Rats (bottom) point of views. The black lines are depth contours at 50 m intervals, starting at 50 m deep. Predicted standard deviation maps are available in Figure S19. Grey background indicates predicted absence. White background indicates Egmont Atoll shape. Egmont is 30 km^2^ (Harris et al. 2024).

**FIGURE 5:**
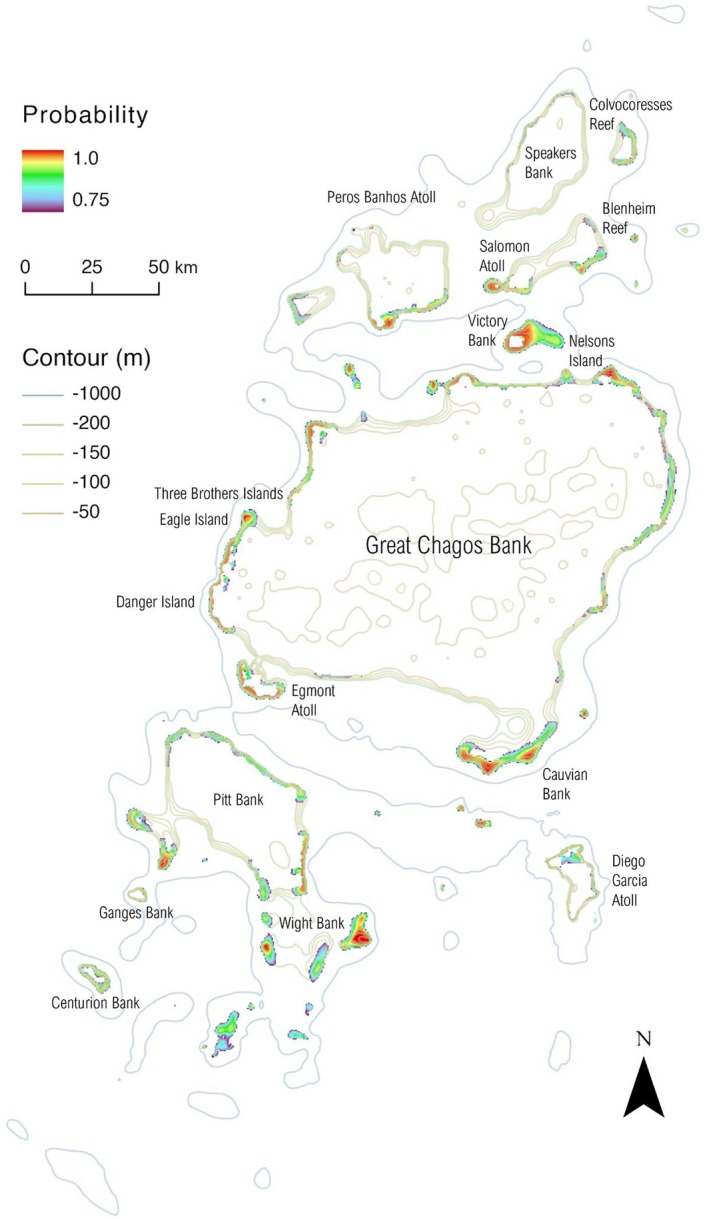
Spatial predictions for MCEs (30–160 m) – low resolution bathymetry cluster for the Chagos Archipelago using the MaxEnt modelling technique. Depth contours of different colours are at 50 m intervals, starting at 50 m deep to 200 m, then showing the 1000 m depth contour. Predicted standard deviation maps are available in Figure S21.

For MCE clusters, topographically derived variables contributed to the best performing models (Table 2). Hence, the probability of occurrence was not as homogeneous around the Atoll as for individual clusters (Figures 4 and 5). Indeed, for the MCE predictions derived from high‐resolution bathymetry data, areas with the highest slope (depicted by the depth contour lines sitting closer together and the 3D bathymetry) and possibly FBPI contained the highest probability of occurrence (Figure 4). Hence, a low probability of MCE occurrence is observed at the southern tip of the Atoll compared to individual clusters (Figures 3, S2 and S4). MCE presence predicted from the low‐resolution bathymetry data indicated that MCEs are predicted to be present all around the Archipelago (Figure 5). However, they are not predicted to occur anywhere with a suitable depth but rather in specific areas where FBPI and BBPI may have specific values (Figure 5).

### 3.3 Model Performance, Evaluation and Validation

All models resulted in excellent (> 0.9) performances for the three threshold‐dependent (MaxSens+Spec; Sens = Spec; MinROCdist) metrics and the threshold‐independent metric (AUC) (Tables S4 and S5, Supporting Information S4), resulting in thresholds ranging from 0.18 to 0.37 for the distinct benthic clusters (1 to 6); 0.22 for the MCE cluster derived from high‐resolution bathymetry data; and 0.75 for the MCE cluster derived from low‐resolution bathymetry data. These threshold values were chosen to optimize the model performance. The three threshold‐dependent, as well as the threshold‐independent metrics, revealed fair to excellent (> 0.7 to > 0.9) performances for test and train models (Tables S4 and S5, Supporting Information S4). The average nearest neighbor test revealed that the image data were highly clustered (nearest neighbour ratio: 0.16; *z*‐score: −32.8; *p*‐value: < 0.001). Results of the independent validation suggest that the two MCE original models performed worse than expected based on results for both threshold‐dependent and ‐independent metrics (Tables S4 and S5). Nevertheless, the model performance for the high‐resolution MCE model is considered fair (0.7–0.8), with poorer performance for the low‐resolution MCE model (Table S4). The high‐resolution model was better able to predict the absences (specificity) than the presences (sensitivity), while the low‐resolution model predictions varied in specificity/sensitivity performance depending on the threshold chosen (Table S5). The correctly and incorrectly predicted presence/absence can also be seen in the confusion matrix (Table S6).

### 3.4 Comparison of Predicted Extent and Distribution for High Versus Low Resolution Models

The spatial extent for the two MCE models varied between the two data resolutions. Using high‐resolution bathymetric data, MCEs covered an area of only 3.1% of that modelled using low‐resolution bathymetry data, at Egmont Atoll specifically (2.55 vs. 82.4 km^2^ for high‐ and low‐resolution data, respectively, Table 3). When expanded to the entire Archipelago, MCE habitats are predicted to cover 2297.2 km^2^ calculated with GEBCO bathymetry (low‐resolution), compared to 71.1 km^2^ of cover with the multibeam data (high‐resolution) (Table 3). However, due to the extreme difference in topography between satellite‐derived GEBCO data and multibeam survey data, the predictions of MCE presence/absence will respond differently to the topography at these two different scales (Supporting Information S6). In terms of distribution, high‐ and low‐resolution models are broadly similar at Egmont Atoll, with higher MCE predictions on the western part of the Atoll. Additionally, differences in terms of the spatial extent of MCEs predicted from the models can be observed between MCE low/high‐resolution bathymetry data and the predicted clusters summed together (individual clusters from 30 to 160 m). A total of 9.42 km^2^ of suitable habitat is predicted at Egmont Atoll based on the predicted clusters summed together, corresponding to 11.4% of the area predicted using low‐resolution data and 369.5% of the area predicted using high‐resolution data (Table 3). Hence, summed clusters differ from the entire MCE models in terms of probability of cover, with a predicted cover of 262.8 km^2^ over the entire Archipelago (Table 3). Finally, the independent validation data set revealed that both high‐ and low‐resolution MCE models incorrectly predicted absences more than presences (Table S6), potentially underestimating the extent of MCEs in the Archipelago or not fully capturing the drivers of MCE distribution.

**TABLE 3:** Area of predicted suitable habitat for MCEs in km^2^, for high‐resolution bathymetry data (9 m^2^) and low‐resolution bathymetry data (472 m^2^), and individual clusters (1–6).

Predicted MCEs area (km^2^)	Egmont Atoll	Chagos Archipelago
MCEs – low‐resolution	82.4	2297.2
MCEs – high‐resolution	2.55	71.1^a^
Cluster 1	0.67	—
Cluster 2	1.31	—
Cluster 3	2.95	—
Cluster 4	1.81	—
Cluster 5	1.28	—
Cluster 6	2.07	—
Summed clusters 30–160 m	9.42	262.8^a^

^a^ Calculated based on Egmont Atoll surface extent.

## 4 Discussion

### 4.1 Drivers of Mesophotic Benthic Cluster Distribution

MCEs within the Chagos Archipelago are influenced by a combination of environmental and geomorphological factors, shaping their occurrence and distribution across this region. Among the key environmental drivers, temperature and light (measured as PAR) emerge as strong predictors of both distinct benthic clusters and MCEs as a whole (Table 2). Light and mean temperature showed decreasing responses for clusters of increasing depth, as shown in a parallel study (Diaz et al. 2023c), and temperature variability increased for clusters of increasing depth (Supporting Information S3). These factors, which also serve as proxies for depth and distance from shore (Kleypas et al. 1999), play a pivotal role in determining the prevalence of MCEs, not only in this region but globally, as evident from previous studies (Tamir et al. 2019). In particular, temperature is an important factor as it regulates an organism's metabolism and was highly correlated with depth, assessed as a key driver of MCE community changes (Pyle et al. 2016). Temperature variability (i.e., delta) is acting as a proxy for the thermocline, internal waves, and oxygen or nutrient availability (Hosegood et al. 2019; Nelson and Altieri 2019). In this study, PAR was found to contribute to the distribution of all the clusters, except the deepest one (150–160 m), where light was no longer available (Diaz et al. 2023c).

Adding to the complexity, Chl‐a, a proxy for phytoplankton concentration (i.e., primary productivity) transported by currents, but also for thermocline depth, emerged as a significant predictor, particularly for the deeper clusters (3–6) and the high‐resolution MCE cluster (Table 2). As suggested by Swanborn et al. (2022), the relationships between the benthic clusters and their environment appear to be related to their functional characteristics within their ecosystem. A shift from autotrophic organisms such as photosynthetic corals towards heterotrophic organisms such as octocorals and antipatharians occurred at the depth of the deep chlorophyll maximum (DCM) around 60 m (Table 1, Figure 2).

Geomorphology, another critical factor shaping benthic communities (Swanborn et al. 2022), exhibited its influence on the distribution of MCEs, though not as homogeneously as temperature and light. While individual benthic clusters seemed unaffected by geomorphological parameters, they were correlated with significant oceanographic parameters and became important when assessing the broader MCE distribution, at both high‐ and low‐bathymetric resolutions (Table 2). This may be due to the small scale at which individual benthic clusters were distinguished, hence not capturing the geomorphological differences between clusters, unlike MCE clusters that covered a broader scale. Slope, FBPI, and BBPI contributed to the predicted distribution of the MCE clusters, as observed in numerous studies around the world, for example, in the Great Barrier Reef (Bridge et al. 2012); the South‐West Indian Ocean (Swanborn et al. 2022); Hawai'i (Costa et al. 2015); and the northern part of the Gulf of Mexico (Silva and MacDonald 2017; Sterne et al. 2020). Geomorphology has been identified as a key driver not only for MCEs but also for benthic communities associated with hard bottom habitats across all depths (Rengstorf et al. 2013; Swanborn et al. 2022). Indeed, in addition to being an indicator of suitable substrate for reef colonization, geomorphology components such as slope also mediate the hydrodynamic regime encountered by the reef, hence influencing food availability for suspension‐feeding organisms (Bridge et al. 2011; Sterne et al. 2020) or surrounding temperature variability for photosynthetic corals (Diaz et al. 2023a). MCEs in the Chagos Archipelago were predicted to occur in sloped and topographically complex areas rather than following the depth contour (Figures S2 and S4), making geomorphological factors an additional key discriminator in their spatial distribution.

### 4.2 MCE High‐ Versus Low‐Resolution Models and Distribution Across the Chagos Archipelago

In this study, the performance of both high‐ and low‐resolution models was similar (Tables S4 and S5), albeit variations may depend on the species and study area (Rengstorf et al. 2013; Ross et al. 2015). However, the independent validation data sets had better performances for the high‐resolution models compared to the low‐resolution models (Tables S4 and S5), revealing the importance of using independent validation to evaluate spatial distribution models.

Differences in the predicted spatial extent have been repeatedly shown between the two models. The GEBCO‐derived data predicted a vast area of 2297.2 km^2^ occupied by MCEs across the Archipelago, whereas high‐resolution multibeam data predicted a much smaller extent of only 71.1 km^2^, resulting in a large difference of over 96% (Table 3). However, a direct comparison of the extent between the two model predictions cannot realistically be made, as they do not entirely overlap, revealing that the difference in prediction is not only due to the resolution but also to the inaccuracy of the GEBCO data (Supporting Information S6, further discussed in Section 4.3 ‘Methodological considerations’). Moreover, when considering geomorphological parameters in the models, there were further variations in spatial extent predictions.

The summed clusters model (clusters 1–6) predicted a larger area of 262.8 km^2^ for the entire Archipelago compared to the 71.1 km^2^ predicted by the MCE model derived from high‐resolution data (Table 3). The summed clusters were projected to occur all around Egmont Atoll, including where the underwater topography was unfavourable for MCE occurrence (Figures S2 and S4, pers. Obs.), in contrast to the MCE high‐resolution model that predicted MCEs in rather steeper slopes and geomorphologically complex regions (Figure 4). This trend has been highlighted in previous MCE modelling studies (Bridge et al. 2012; Costa et al. 2015; Sterne et al. 2020; Swanborn et al. 2022), emphasising the influential role of geomorphological parameters in shaping MCE distribution. In some cases, these parameters may even be the only factors needed to successfully model MCE distribution over smaller areas (Bridge et al. 2012; Swanborn et al. 2022). Interestingly, the validation data set revealed that the two models—the low‐resolution GEBCO model in particular—may not have overestimated the MCEs’ extent around the Archipelago, in contrast to common observations in deep‐sea (Rengstorf et al. 2013; Howell et al. 2022) or terrestrial studies (Seo et al. 2009). The confusion matrix shows that the models contained a greater number of incorrectly predicted absence points compared to incorrectly predicted presence points (Table S6).

In addition to other caveats discussed in the ‘methodological considerations’ section, MCEs occupy a relatively small depth span (~110 m) compared to deep‐sea ecosystems, and the GEBCO bathymetry resolution used in this study is coarser (472 m) than the MCE depth range, potentially leading to this lack of predicted overestimation in terms of MCE coverage. Whilst the high‐resolution model performed better, it can only be properly used where high‐resolution bathymetry is available. The GEBCO model did not perform as well when independently validated but can estimate MCEs over a larger area, such as the entire Chagos Archipelago in this study.

The current study predicted extensive MCE occurrence across the entire Chagos Archipelago, spanning from the North to the South. MCEs were associated with various geomorphologies (i.e., atolls; islands; submerged banks; reefs drop‐offs; seamounts) with many of these already recognised as suitable MCE habitats (Bridge et al. 2012; Costa et al. 2015; Silva and MacDonald 2017; Sterne et al. 2020; Swanborn et al. 2022). The validation data set and model predictions indicated the presence of MCEs in every atoll (Egmont, Salomon, Peros Banhos and Diego Garcia Atolls), every island (Three Brothers, Eagle, Danger, Nelsons Islands), and every submerged bank (Great Chagos, Cauvian, Pitt, Wight, Centurion, Ganges, Victory, and Speakers Banks) identified within the Archipelago (Figures 1 and 5). In addition, submerged reefs (Blenheim and Colvocoresses reefs) and seamounts were identified as MCE habitats, demonstrating the wide‐ranging distribution of these unique ecosystems (Figures 1 and 5).

### 4.3 Methodological Considerations

Extensive sampling of MCEs is challenging, as they lie beyond recreational SCUBA depth. In particular, the difficulty in accessing the Chagos Archipelago and constrained time on site limited the spatial area covered in this study across the full MCE depth range, resulting in a scarcity of presence/absence data points covering a large spatial area within the Archipelago, nevertheless needed to build models of high quality. The reduced number of presence points is further emphasiszed by MCE predictions from low‐resolution bathymetry and may have influenced the cluster separation in the first instance. In addition, the models displayed in this study are based on two sites in Egmont Atoll for the biological data, which may not be representative of the wider Archipelago in terms of mesophotic benthic communities. Nevertheless, temperature characteristics, salinity, chl‐a, and PAR variables were collected at several sites and over several years to allow for better confidence in the extrapolations made in this study. The long‐term environmental data extracted from Argo floats and averaged over four years may have yet failed to account for the differences between benthic communities over the slopes and in the open ocean in terms of spatial and temporal variations. Spatial proxies such as distance to shore were not included here, unlike other studies, but considered as highly correlated to depth and light (Costa et al. 2015; Swanborn et al. 2022). This region remains extremely complex in terms of bathymetry and oceanography, with potential scaling issues in the models that may have been amplified using GEBCO data. Therefore, making perfect predictions of a given region based on a subsample of locations is not possible.

Due to the small number of data points, spatial autocorrelation could not be controlled (either by removing spatially clustered localities, e.g. Spiers et al. (2018), or by separating the data per transect during model evaluation e.g. Howell et al. (2022)). The high spatial autocorrelation found in this study may have inflated model performance values (Brown 2014), in addition to being a reason for the relatively poor performance of the low‐resolution model when using the independent validation dataset. Autocorrelation is possibly an important factor that may have affected our results; however, there is no ‘standard’ and well‐explored method to address it as yet, and it is a largely unresolved issue in species distribution modelling (Dormann et al. 2007). The MCE models here used background points instead of absence points, which may have also resulted in inflated performance values. In terms of performance assessment, the Brier score and McFadden R^2^ were not considered in this study but have previously displayed almost identical results as the AUC (Venne and Currie 2021).

Finally, unlike the validation results, the coverage extent of MCEs across the Chagos Archipelago may have been overestimated. Indeed, during the data point selection for the low‐resolution model (please see the methods section), a low‐resolution grid cell was set as “presence” when > 50% of the high‐resolution points were set as “presence” with consequently potential “absence” points ignored in the same cell. In addition, a different (lower) threshold value would have led to a different low‐resolution extent estimation of MCE coverage, but here the threshold chosen to optimize the model included all the presence points. In general, while being a good tool for benthic community visualization and highlighting areas for further investigation, caution is required when predictive maps are to be utilised in marine spatial management (Lecours 2017; Swanborn et al. 2022), especially when the original dataset is limited.

### 4.4 Conservation Implications and Conclusion

Modelling the distribution of MCEs is still in its infancy, as some key knowledge gaps, such as assemblage composition information, and drivers of occurrence and distribution, are limiting their implementation (Swanborn et al. 2022). Our study contributed to filling some of the knowledge gaps by identifying environmental drivers of MCE occurrence and distribution around Egmont Atoll and the Chagos Archipelago, an area covering 640,000 km^2^ in the central Indian Ocean, as well as distinct benthic communities forming these ecosystems. Similarly, the few studies that have modelled MCE distribution identified environmental proxies that lead to high model performances (Bridge et al. 2012; Costa et al. 2015; Sterne et al. 2020; Swanborn et al. 2022).

Habitat suitability models are particularly useful in predicting ecosystem distribution where direct in situ observations are challenging or are otherwise not feasible given the time and funding constraints. Indeed, model outputs are useful tools for marine managers to effectively target ecologically important areas for conservation as well as a basis for planning future areas of investigation and target sampling effort (Bridge et al. 2012; Rengstorf et al. 2013; Costa et al. 2015; Sterne et al. 2020; Howell et al. 2022). In this study, underwater banks present all over the Chagos Archipelago as well as Salomon and Egmont Atolls, Southern Peros Banhos, and Western Great Chagos Bank appeared to be important areas for MCEs (Figure 4). However, environmental data, such as topography data derived from satellites in particular, are particularly inaccurate, and further data acquisition is urgently needed in the region and worldwide, as highlighted by the Seabed 2030 initiative (https://seabed2030.org), to adequately predict marine ecosystem occurrence and distribution, and ultimately transfer predictions to other parts of the world.

In conclusion, this study provides the first prediction of the distribution of MCEs and their distinct benthic communities in the Chagos Archipelago, an area covering 640,000 km^2^ in the middle of the Indian Ocean. In particular, it has highlighted the significance of MCEs in terms of coverage extent and response to various environmental factors. In addition, the utilization of habitat suitability modelling has furthered our understanding of the factors influencing MCE occurrence and distribution. MCEs host diverse and endemic benthic and pelagic species (Diaz et al. 2024), and their preservation within the Chagos MPA holds broader significance as a “crossroads” connecting the East and West parts of the Indian Ocean (Sheppard et al. 2012). Finally, this study could support decision making for prioritising future survey sites to study MCEs across the Archipelago. The insights gained from this research hold significant implications for marine spatial planning and the conservation of these remarkable ecosystems, which remain vulnerable to the impacts of climate change and human activities.

## Author Contributions

**Clara Diaz:** conceptualization (equal), data curation (lead), formal analysis (lead), investigation (equal), methodology (equal), writing – original draft (lead). **Nicola L. Foster:** conceptualization (equal), formal analysis (supporting), funding acquisition (equal), investigation (equal), supervision (lead), writing – review and editing (equal). **Edward Robinson:** formal analysis (supporting), writing – review and editing (equal). **Kyran P. Graves:** formal analysis (supporting), methodology (supporting), writing – review and editing (equal). **Kerry L. Howell:** conceptualization (equal), funding acquisition (equal), investigation (supporting), methodology (equal), supervision (supporting), writing – review and editing (equal). **Phil Hosegood:** funding acquisition (lead), project administration (lead), writing – review and editing (equal). **Adam Bolton:** formal analysis (supporting).

## Conflicts of Interest

The authors declare no conflicts of interest.

## Supporting information


Data S1.

## Data Availability

Raw data are available on Dryad, doi: https://datadryad.org/stash/share/ECYrh6dpI‐mtRg7JR4lrwUBhUR‐qLJIKDB5YBugoyp0 Further data information for each image can be found here: https://doi.org/10.5061/dryad.wh70rxwxg. The benthic species catalogue is currently on Zenodo (Diaz et al. 2023b).
