# Supplementary material for: Predicting the Distribution of Mesophotic Coral Ecosystems in the Chagos Archipelago
Source: Ecol Evol. 2025 Apr 2;15(4):e71130. doi: 10.1002/ece3.71130 (PMC11962199; doi:10.1002/ece3.71130)
Supplement: Supplementary file 1 — Data S1. [file ECE3-15-e71130-s001.docx]

SUPPLEMENTARY MATERIALS

## S.1 Cluster identification

Figure S.1 : Cluster dendrogram from the Similarity Profile Analysis (SIMPROF) routine on the assemblage composition of each transect of the two study sites. Ile Des Rats (IDR) and Manta Alley (MA) revealed 15 statistically significant biological assemblages, with SIMPROF groups labelled from “a” to “o” (refer to Table 1 for cluster descriptions). Dashed line indicates 55% similarity. The grouped clusters selected are shown within the dashed boxes.


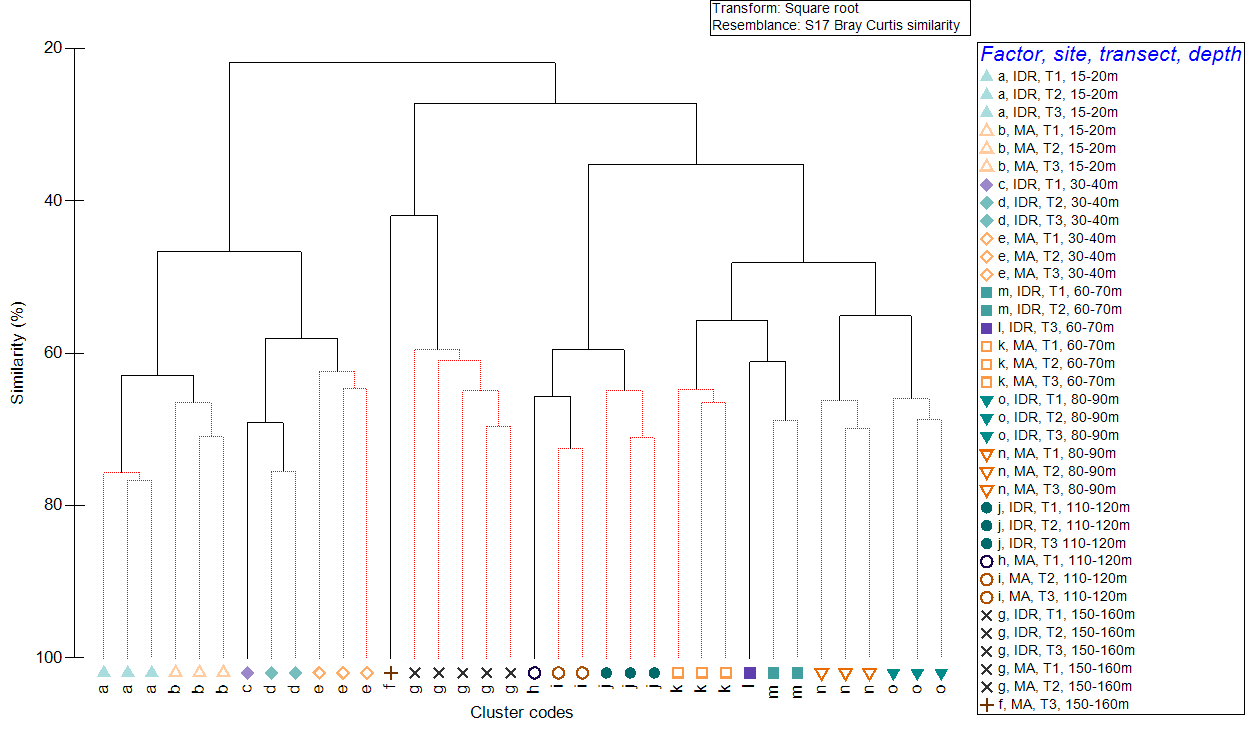


55

Cluster 1

Cluster 2

Cluster 3

Cluster 4


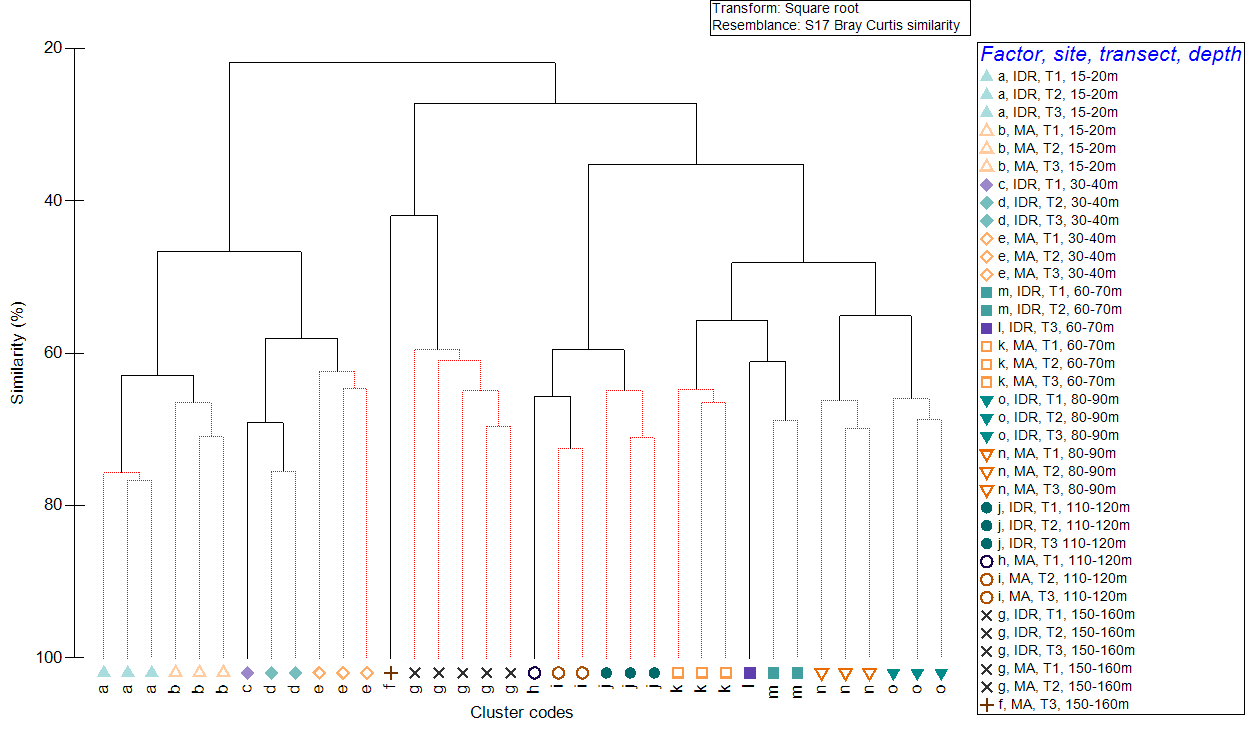


Cluster 6

Cluster 5

Cluster 1

Cluster 2

Cluster 3

Cluster 4

Cluster 5

Cluster 6

## S.2 Variable pre-selection and model performance

Table S.1: Pearson correlation coefficient calculated between environmental variables for the high-resolution bathymetry data. Bathy: depth; BBPI & FBPI: broad & fine-scale benthic position index; Chl-a: chlorophyll-a; PAR: photosynthetically active radiation; Sal: salinity; Rug: rugosity; T min, mean, max, delta: temperature minimum, average, maximum, variability (max-min). Highly correlated variables as well as retained environmental variables are in bold.

|  | Bathy | BBPI | **FBPI** | **Chl-a** | **PAR** | Sal | **Rug** | **Slope** | T min | **T mean** | **T max** | **T delta** |
| --- | --- | --- | --- | --- | --- | --- | --- | --- | --- | --- | --- | --- |
| Bathy | X | - | - | - | - | - | - | - | - | - | - | - |
| BBPI | **0.98** | X | - | - | - | - | - | - | - | - | - | - |
| **FBPI** | 0.62 | 0.61 | X | - | - | - | - | - | - | - | - | - |
| **Chl-a** | 0.42 | 0.43 | 0.27 | X | - | - | - | - | - | - | - | - |
| **PAR** | 0.73 | 0.7 | 0.5 | -0.25 | X | - | - | - | - | - | - | - |
| Sal | -0.8 | -0.78 | -0.48 | 0.14 | **-0.93** | X | - | - | - | - | - | - |
| **Rug** | 0.34 | 0.37 | 0.6 | 0.19 | 0.24 | -0.3 | X | - | - | - | - | - |
| **Slope** | -0.09 | -0.13 | 0.04 | -0.2 | 0.01 | 0.07 | -0.09 | X | - | - | - | - |
| T min | **0.94** | **0.92** | 0.59 | 0.11 | 0.9 | **-0.95** | 0.33 | -0.08 | X | - | - | - |
| **T mean** | **0.99** | **0.97** | 0.6 | 0.55 | 0.63 | -0.72 | 0.35 | -0.12 | 0.88 | X | - | - |
| **T max** | 0.83 | 0.82 | 0.53 | 0.6 | 0.38 | -0.38 | 0.23 | 0 | 0.62 | 0.83 | X | - |
| **T delta** | -0.46 | -0.44 | -0.28 | 0.38 | -0.8 | 0.87 | -0.2 | 0.1 | -0.72 | -0.39 | 0.09 | X |

Table S.2: Pearson correlation coefficient calculated between environmental variables for the low-resolution bathymetry data. Bathy: depth; BBPI & FBPI: broad & fine-scale benthic position index; Chl-a: chlorophyll-a; PAR: photosynthetically active radiation; Sal: salinity; Rug: rugosity; T min, mean, max, delta: temperature minimum, average, maximum, variability (max-min). Highly correlated variables as well as retained environmental variables are in bold.

|  | Bathy | **BBPI** | **FBPI** | **Chl-a** | **PAR** | Sal | Rug | Slope | T min | **T mean** | T max | **T delta** | |
| --- | --- | --- | --- | --- | --- | --- | --- | --- | --- | --- | --- | --- | --- |
| Bathy | X | - | - | - | - | - | - | - | - | - | - | - |  |
| **BBPI** | **0.93** | X | - | - | - | - | - | - | - | - | - | - |  |
| **FBPI** | -0.63 | -0.31 | X | - | - | - | - | - | - | - | - | - |  |
| **Chl-a** | 0.48 | 0.69 | 0.2 | X | - | - | - | - | - | - | - | - |  |
| **PAR** | **0.92** | 0.76 | -0.77 | 0.13 | X | - | - | - | - | - | - | - |  |
| Sal | -0.79 | -0.52 | **0.94** | 0.01 | **-0.91** | X | - | - | - | - | - | - |  |
| Rug | 0.25 | 0.49 | 0.41 | 0.9 | -0.08 | 0.24 | X | - | - | - | - | - |  |
| Slope | -0.87 | -0.65 | **0.91** | -0.13 | **-0.93** | **0.97** | 0.16 | X | - | - | - | - |  |
| T min | **0.92** | 0.73 | -0.84 | 0.24 | **0.95** | **-0.96** | 0 | **-0.97** | X | - | - | - |  |
| **T mean** | **0.98** | 0.9 | -0.65 | 0.55 | 0.88 | -0.8 | 0.3 | -0.88 | **0.93** | X | - | - |  |
| T max | **0.91** | **0.96** | -0.35 | 0.54 | 0.75 | -0.5 | 0.37 | -0.65 | 0.69 | 0.85 | X | - |  |
| **T delta** | 0.02 | 0.32 | 0.6 | 0.38 | -0.22 | 0.56 | 0.47 | 0.38 | -0.36 | -0.08 | 0.42 | X |  |


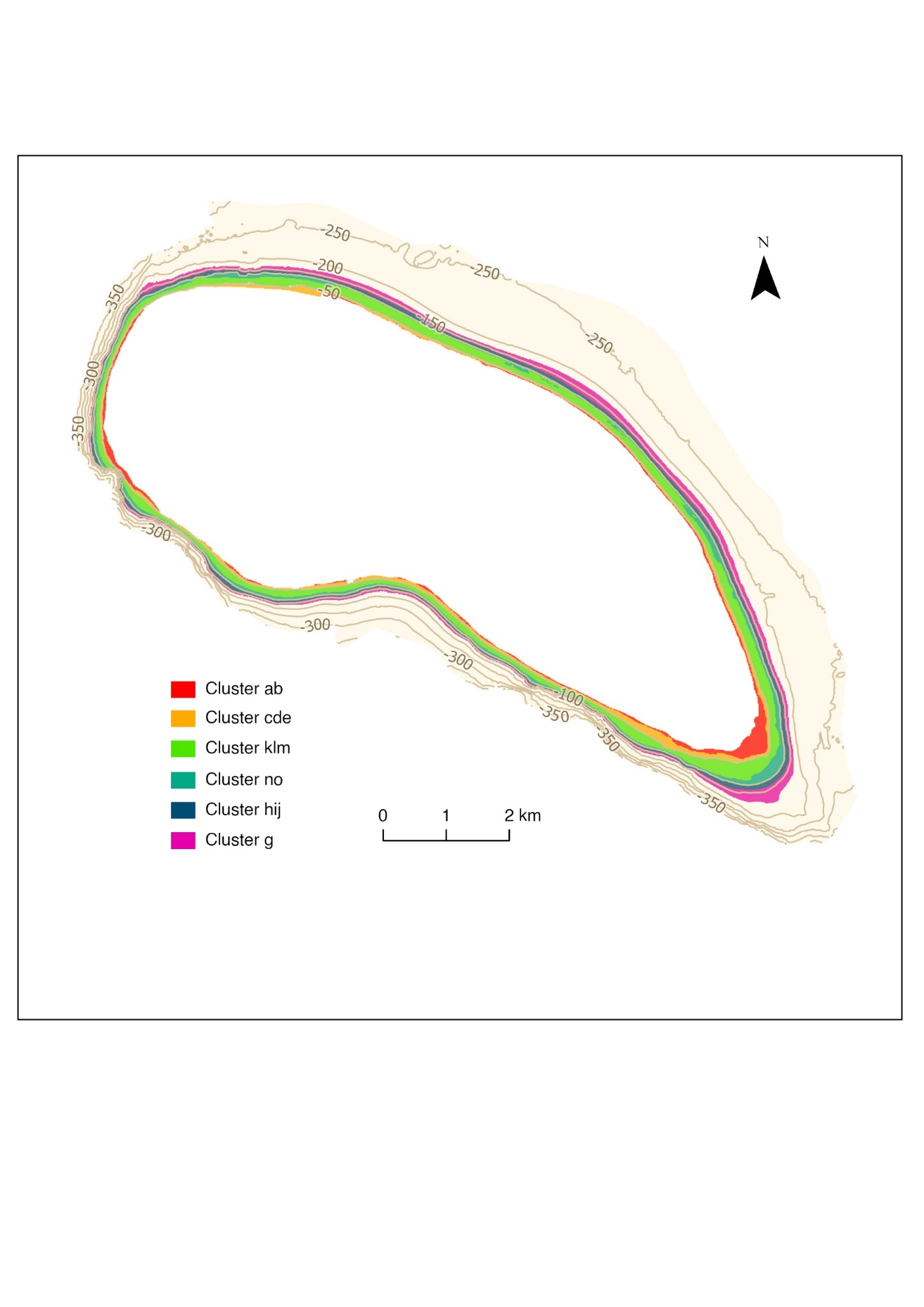


1

2

3

4

5

6

Figure S.2: Binary illustration of each cluster assemblage (1 to 6) at Egmont Atoll using MaxEnt modelling technique, when solely based on depth. Coloured colours indicate presence whilst beige colour indicates predicted absence. White background indicates Egmont Atoll shape.

Table S.3: Biological datasets per assemblage cluster (grouping Ile Des Rats and Manta Alley) showing total and presence/absence points to build and validate the models. Presence/ absence corresponds to the number of images containing/lacking the cluster and data points used for subsequent analysis. MCEs cluster using high/low-resolution bathymetry data. The collected model validation datasets correspond to the number of images collected during the expedition and reduced to one point per cell. The retained datasets correspond to the number of images that fell into the original model domains.

| **Cluster** | **Presence** | **Absence** | **Total** |
| --- | --- | --- | --- |
| 1 | 39 | 381 | 420 |
| 2 | 67 | 353 | 420 |
| 3 | 88 | 332 | 420 |
| 4 | 71 | 349 | 420 |
| 5 | 74 | 346 | 420 |
| 6 | 66 | 354 | 420 |
| MCEs (high-resolution) | 381 | 39 | 420 |
| MCEs (low-resolution) | 12 | 1 | 13 |
| **Model validation datasets** | | | |
| MCEs (high-resolution) | 33 | 38 | 71 |
| MCEs (low resolution) | 14 | 13 | 27 |
|  |  |  |  |

Table S.4: Mean (standard deviation) area under the receiver operating characteristic (ROC) curve (AUC) for the training, test and full models, for each identified cluster. MCEs high/low: MCE cluster from high/low resolution bathymetry data, 3 decimal points are displayed. Validation: MCEs -high/low are the metrics obtained from the independent validation data points, based on binary predictions (not partitioned as test/train data).

| **Cluster** | **AUC test** | **AUC train** | **AUC full model** |
| --- | --- | --- | --- |
| **1** | 0.879 (0.024) | 0.999 (0.001) | 0.999 (0.009) |
| **2** | 1 (0) | 0.999 (0.001) | 0.999 (0.001) |
| **3** | 0.896 (0.019) | 0.999 (0) | 0.999 (0) |
| **4** | 0.999 (0.001) | 0.999 (0.001) | 0.999 (0.001) |
| **5** | 1 (0) | 1 (0) | 1 (0) |
| **6** | 0.990 (0.007) | 0.985 (0.006) | 0.986 (0.005) |
| **MCEs – high** | 0.973 (0.009) | 0.974 (0.002) | 0.974 (0.002) |
| **Validation: MCEs – high** | - | - | 0.738 |
| **MCEs – low** | 0.992 (NA) | 0.994 (0.002) | 0.994 (0.002) |
| **Validation: MCEs – low** | - | - | 0.602 |

Table S.5: Threshold-dependent evaluation metrics for training, test and full models. Chosen threshold method is MaxSens+Spec (sensitivity-specificity sum maximization). Values given are means followed by standard deviation given in brackets. Thres: threshold values, which are the predicted probabilities of presence. PCC: percent correctly classified; Sens: sensitivity; Spec: specificity; MCEs high/low: MCE cluster from high/low resolution bathymetry data. MCEs -high/low are also shown with validation metrics obtained from the independent validation data points, for the original and new thresholds (which were not partitioned as test/train data).

| **Cluster** | **Method** | **Average test** | | | **Average training** | | | **Full model** | | | | **Thres** | |  |
| --- | --- | --- | --- | --- | --- | --- | --- | --- | --- | --- | --- | --- | --- | --- |
|  |  | **PCC** | **Sens** | **Spec** | **PCC** | **Sens** | **Spec** | **PCC** | **Sens** | **Spec** |  | |  |  |
| **1** | MaxSens+Spec | 0.960 (0.010) | 0.710 (0.010) | 0.990 (0.010) | 0.980 (0.010) | 1 (0) | 0.970 (0.010) | 0.97 (0.010) | 1 (0) | 0.970 (0.010) | 0.18 | |  |  |
|  |  |  |  |  |  |  |  |  |  |  |  |  |  | |
| **2** | MaxSens+Spec | 0.999 | 1 (0) | 0.999 | 0.995 | 1 (0) | 0.990 (0.004) | 0.995 (0.003) | 1 (0) | 0.990 (0.004) | 0.31 | |  | |
|  |  | -0.001 |  | -0.001 | -0.004 |  |  |  |  |  |  |  |  | |
| **3** | MaxSens+Spec | 0.901 | 0.842 | 0.922 (0.021) | 0.989 (0.006) | 1 (0) | 0.985 (0.008) | 0.988 (0.005) | 1 (0) | 0.985 (0.007) | 0.225 | |  | |
|  |  | -0.016 | -0.029 |  |  |  |  |  |  |  |  |  |  | |
| **4** | MaxSens+Spec | 0.99 | 1 (0) | 0.988 | 0.987 | 1 (0) | 0.985 | 0.988 | 1 (0) | 0.986 | 0.19 | |  | |
|  |  | -0.007 |  | -0.009 | -0.008 |  | -0.009 | -0.005 |  | -0.006 |  |  |  | |
| **5** | MaxSens+Spec | 1 (0) | 1 (0) | 1 (0) | 1 (0) | 1 (0) | 1 (0) | 1 (0) | 1 (0) | 1 (0) | 0.37 | |  | |
| **6** | MaxSens+Spec | 0.975 | 1 (0) | 0.97 | 0.964 (0.011) | 1 (0) | 0.957 (0.013) | 0.967 (0.009) | 1 (0) | 0.96 | 0.24 | |  | |
|  |  | -0.013 |  | -0.016 |  |  |  |  |  | -0.01 |  |  |  | |
| **MCEs-high** | MaxSens+Spec | 0.921 (0.003) | 0.974 (0.023) | 0.921 (0.003) | 0.918 (0.003) | 0.964 (0.010) | 0.916 (0.003) | 0.915 (0.003) | 0.969 (0.009) | 0.913 (0.003) | 0.22 | |  | |
| **MCEs-high** | MaxSens+Spec | Independent validation with original threshold | | | | | | 0.69 (0.055) | 0.612 (0.070) | 0.864 (0.075) | | 0.22 | |  |
| **MCEs-high** | MinROCdist | Independent validation with new threshold | | | | | | 0.718 (0.054) | 0.653 (0.069) | 0.864 (0.075) | | 0.18 | |  |
| **MCEs-low** | MaxSens+Spec | 0.992 | 1 (NA) | 0.992 (0.001) | 0.989 | 0.933 (0.041) | 0.989 (0.001) | 0.990 (0.001) | 1 (0) | 0.990 (0.001) | 0.75 | |  | |
|  |  | -0.001 |  |  | -0.001 |  |  |  |  |  |  |  |  | |
| **MCEs-low** | MaxSens+Spec | Independent validation with original threshold | | | | | | 0.407 (0.096) | 0.421 (0.116) | 0.375 (0.183) | | 0.75 | |  |
| **MCEs-low** | MinROCdist | Independent validation with new threshold | | | | | | 0.444 (0.097) | 0.368 (0.114) | 0.625 (0.183) | | 0.78 | |  |

Table S.6: Confusion matrix of the two MCEs models, showing the predicted true/false presences and absences when compared to the independent validation data set. True positive is the correctly predicted presence, true negative is the correctly predicted absence, false positive is the incorrectly predicted presence and false negative is the incorrectly predicted absence.

| MCEs model | True positive | True negative | False positive | False negative |
| --- | --- | --- | --- | --- |
| High-resolution | 30 | 19 | 3 | 19 |
| Low-resolution | 8 | 3 | 5 | 11 |

## S.3 Final model Jackknife plots and response curves

### S.3.1 Cluster 1


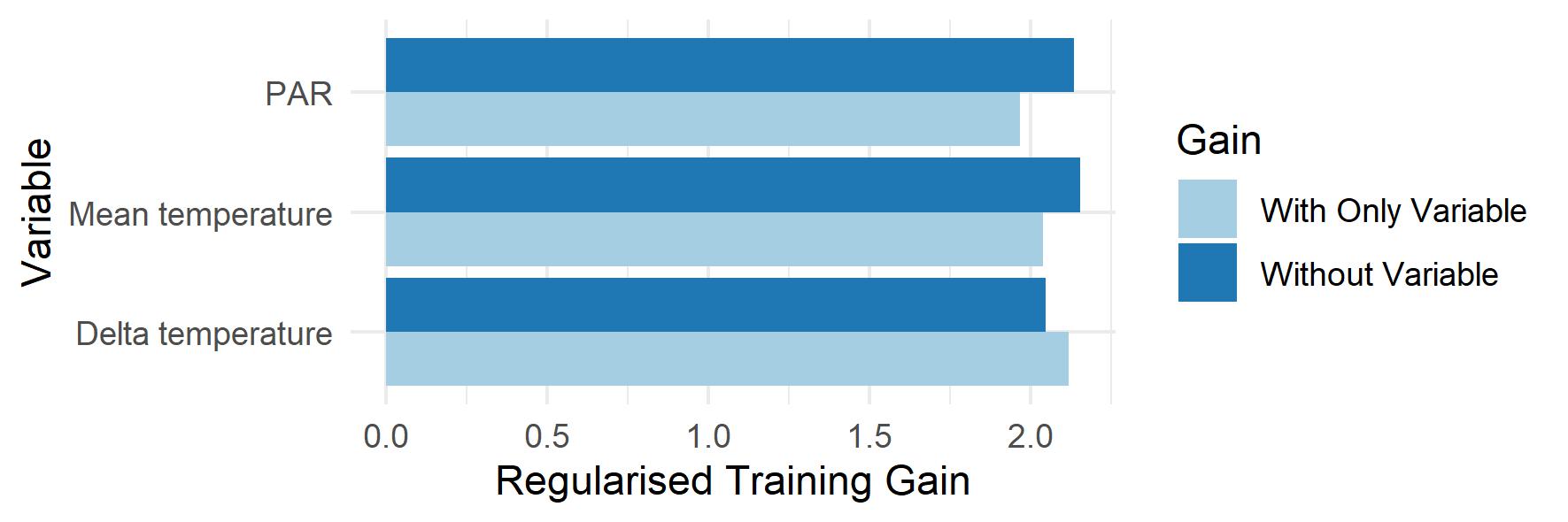


Figure S.3: Jackknife plot of the selected variables, cluster 1.


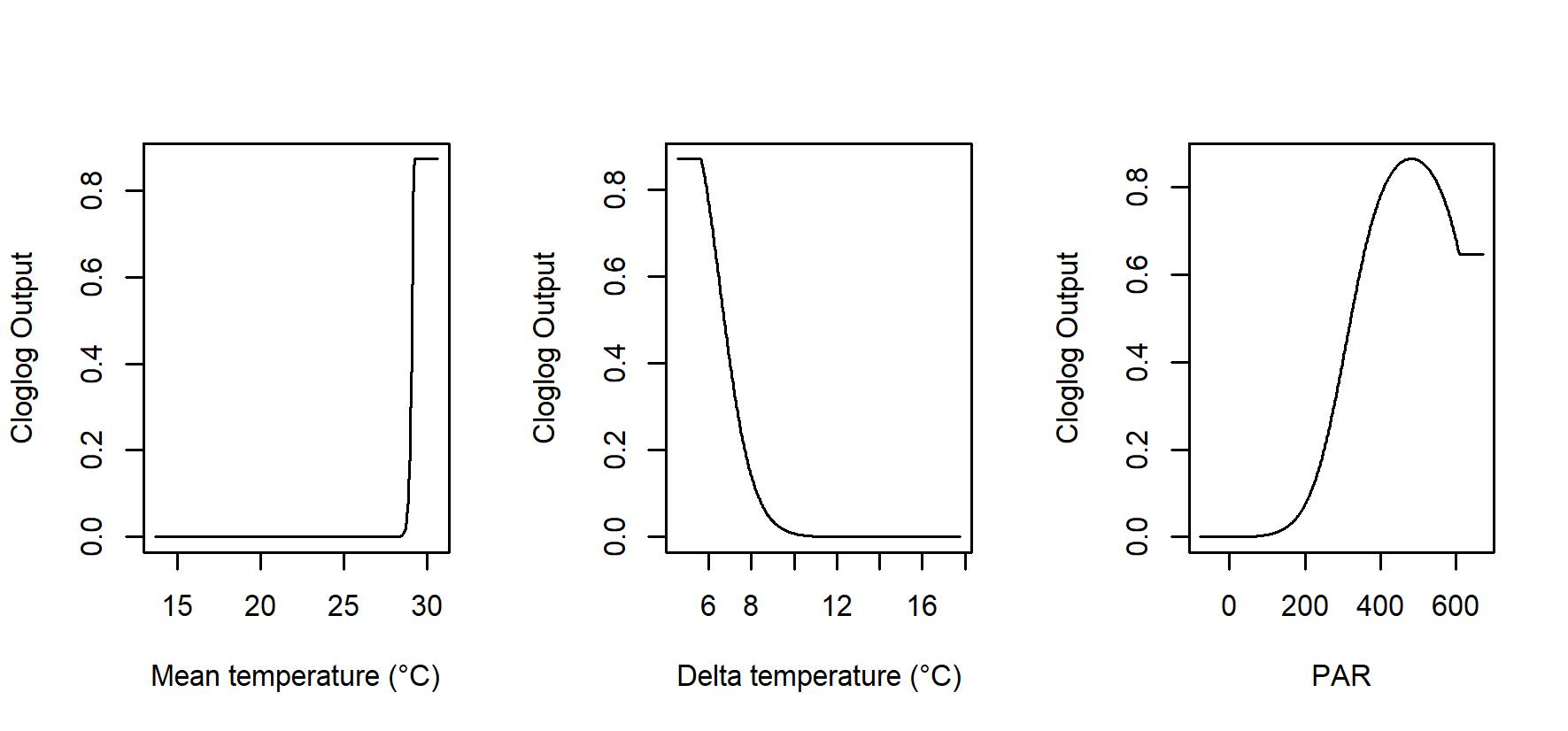


Figure S.4: Response curves after variable selection, cluster 1.

### S.3.2 Cluster 2


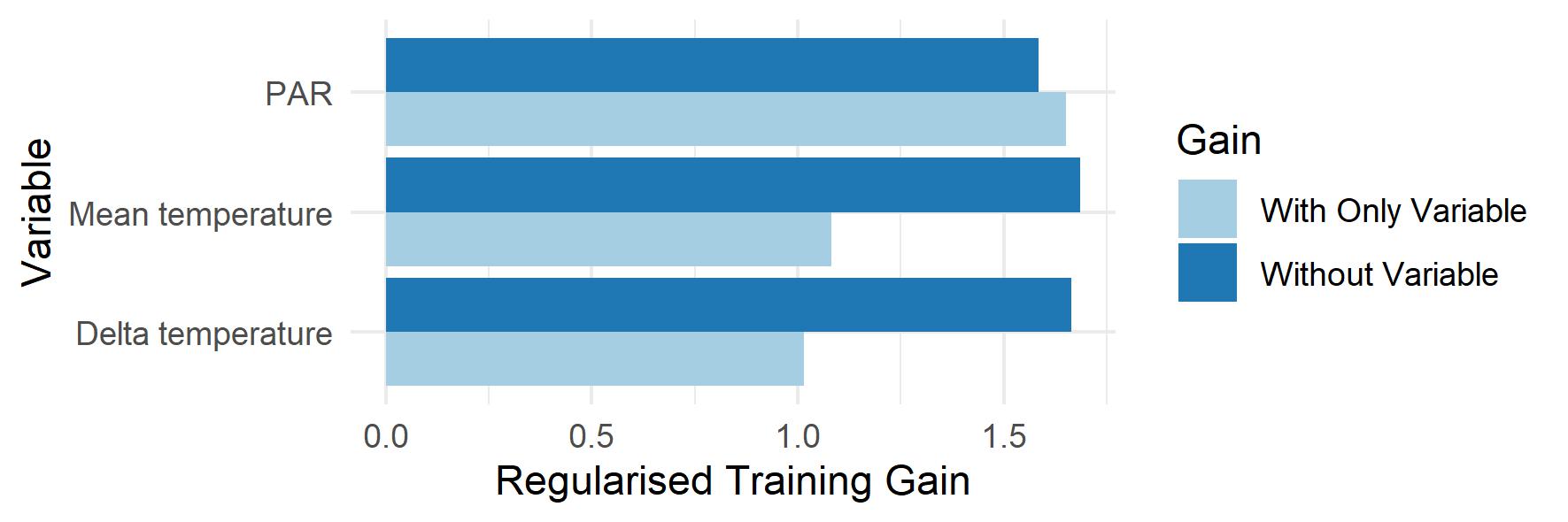


Figure S.5: Jackknife plot of the selected variables, cluster 2.


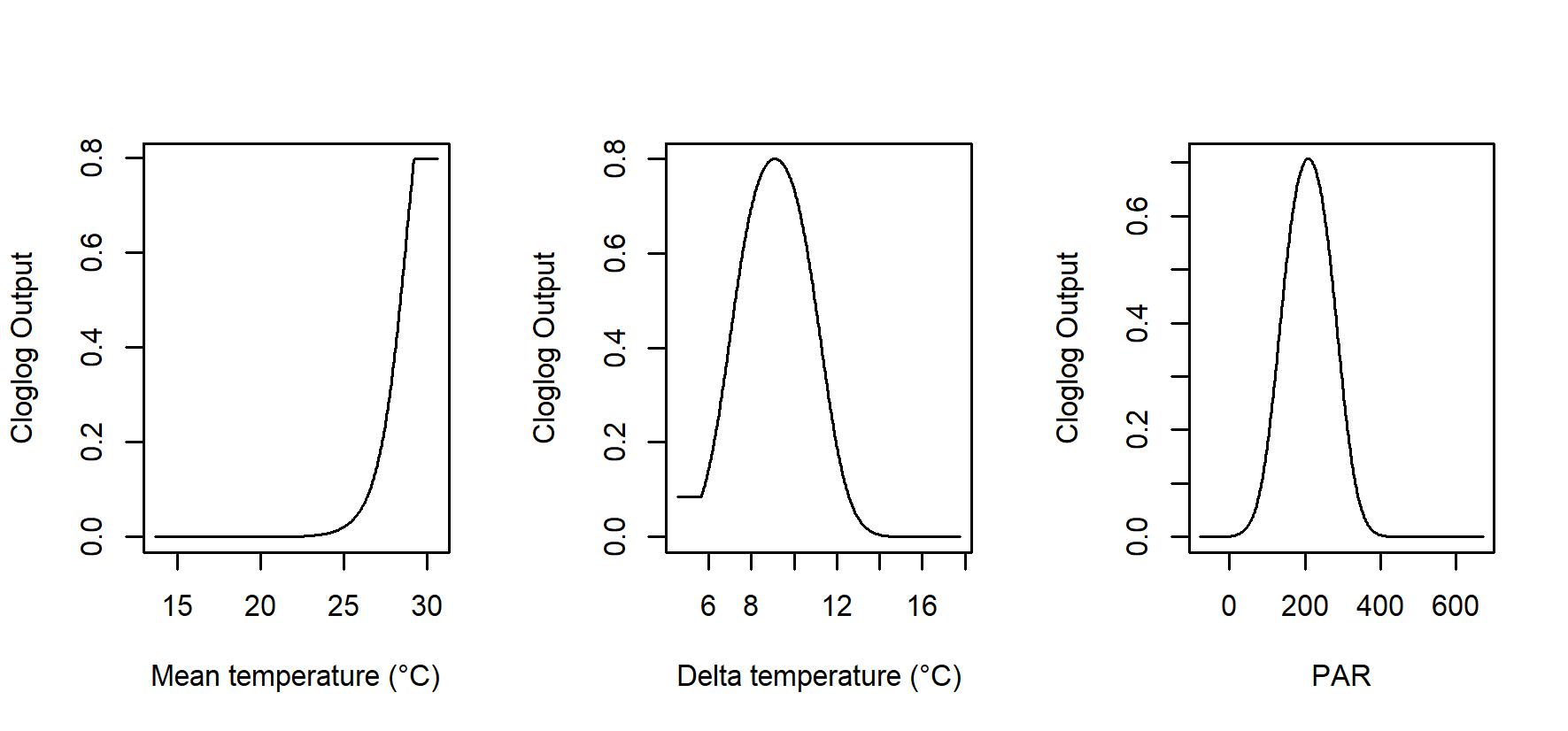


Figure S.6: Response curves after variable selection, cluster 2.

### S.3.3 Cluster 3


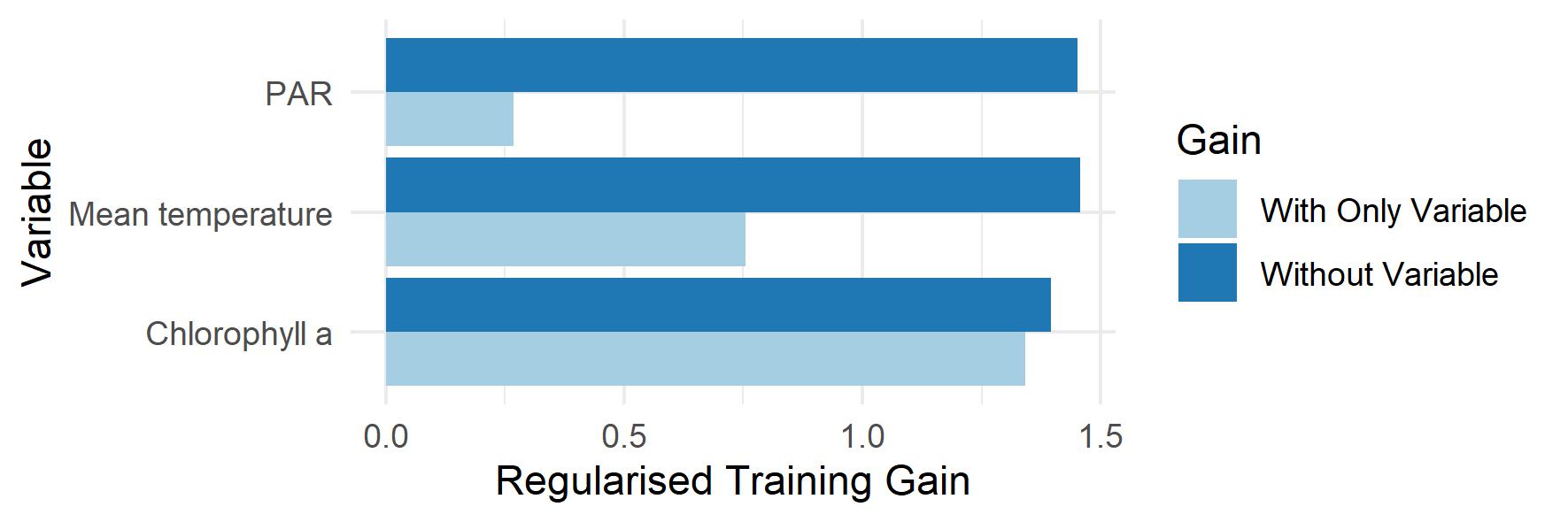


Figure S.7: Jackknife plot of the selected variables, cluster 3.


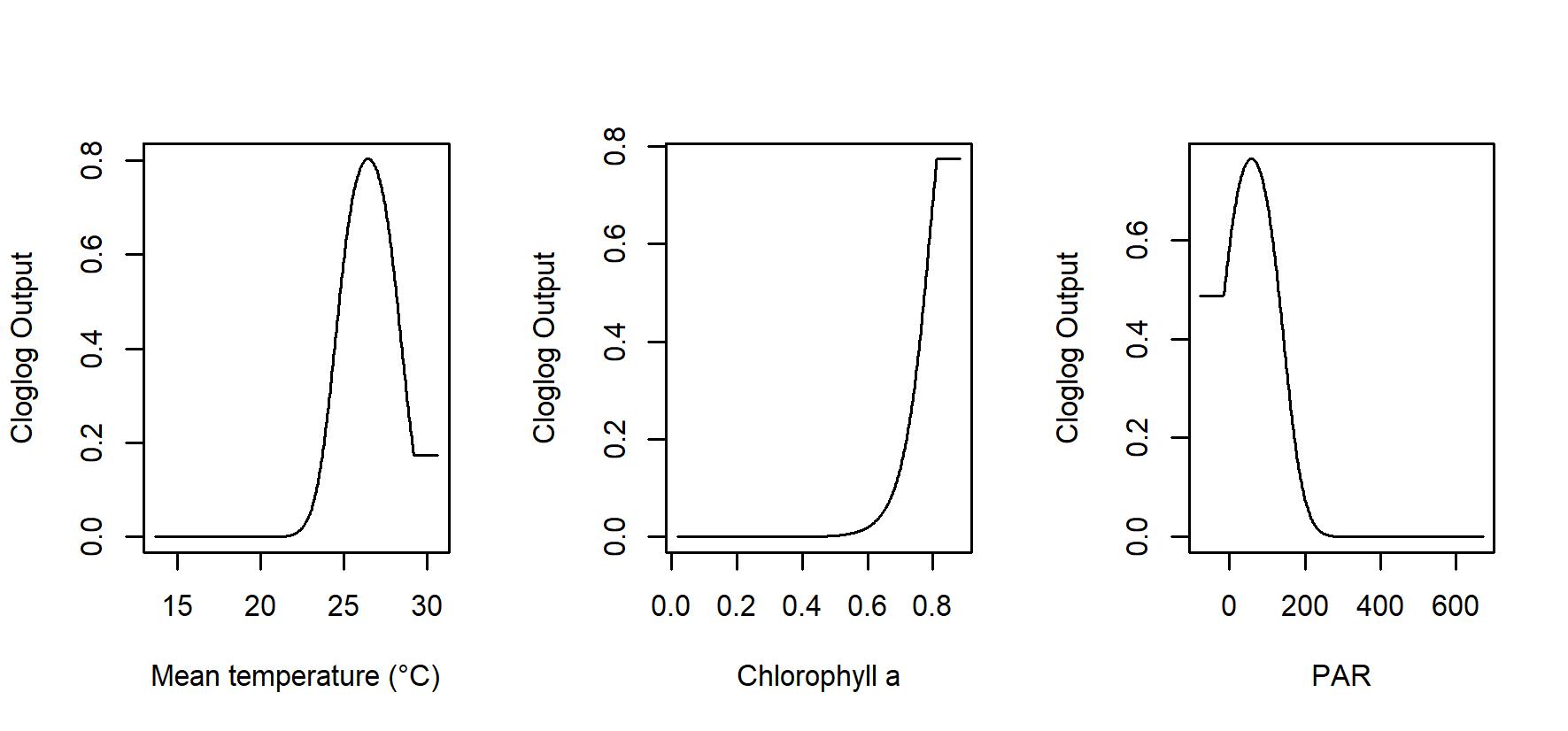


Figure S.8: Response curves after variable selection, cluster 3.

### S.3.4 Cluster 4


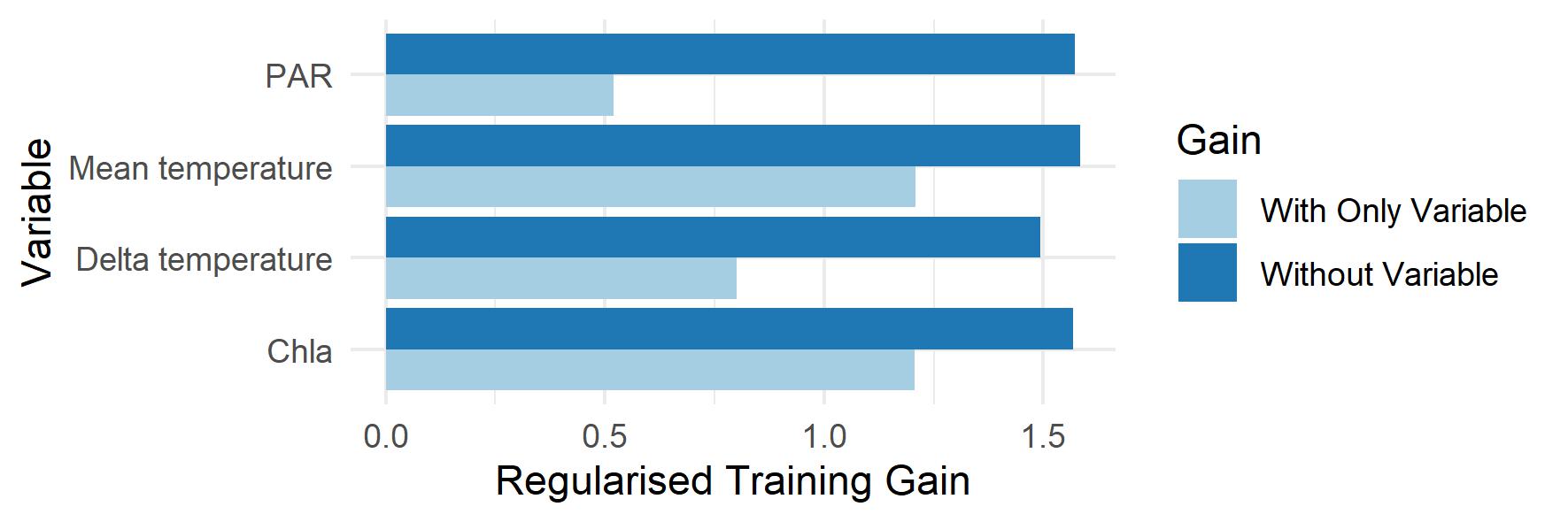


Figure S.9: Jackknife plot of the selected variables, cluster 4.


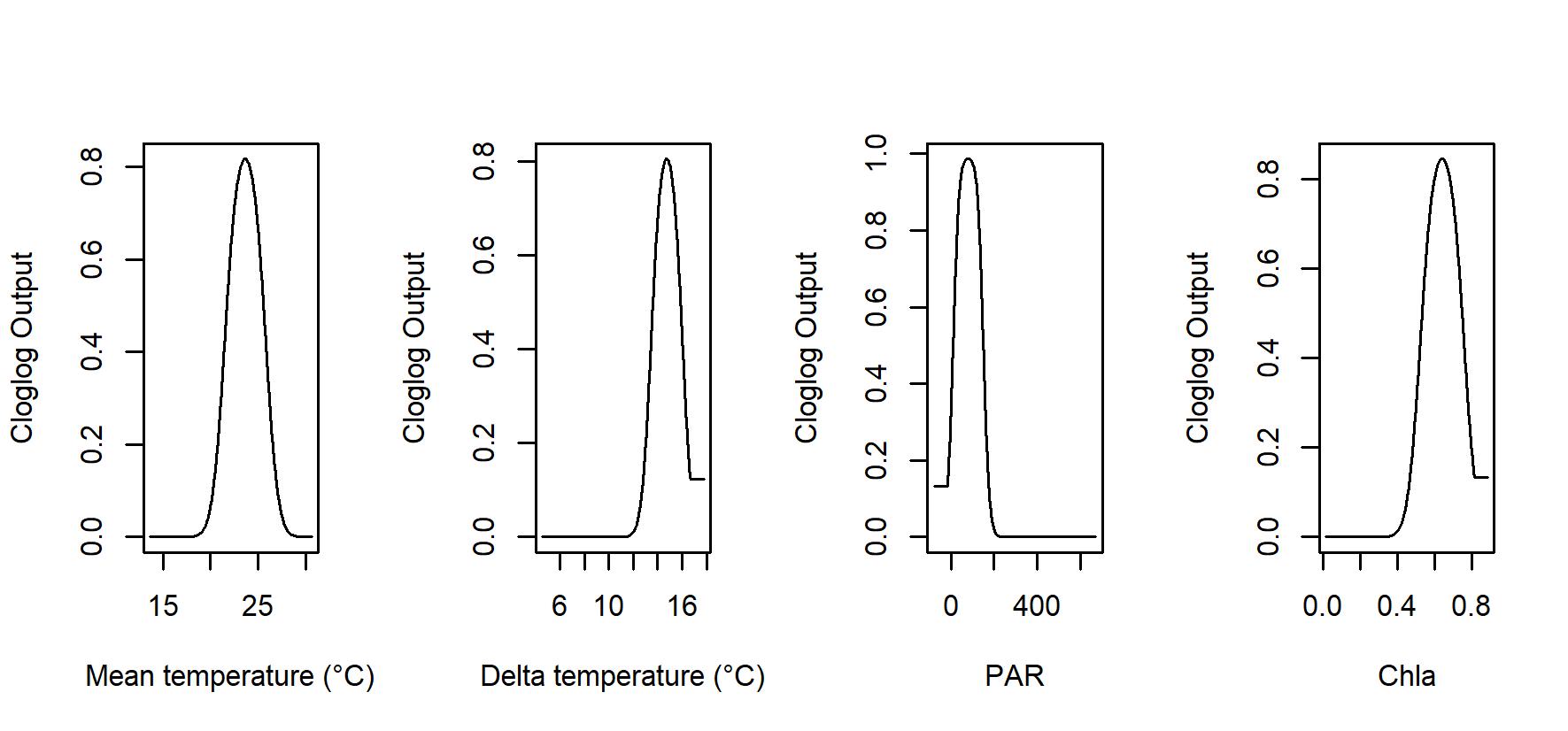


Figure S.10: Response curves after variable selection, cluster 4.

### S.3.5 Cluster 5


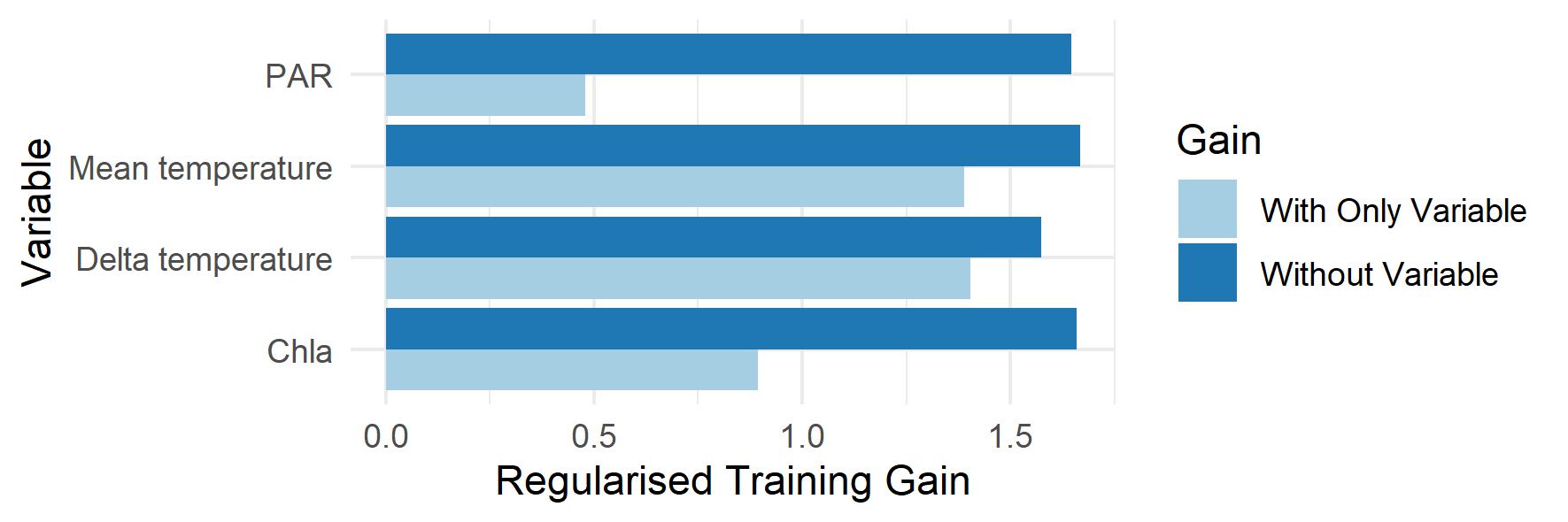


Figure S.11: Jackknife plot of the selected variables, cluster 5.


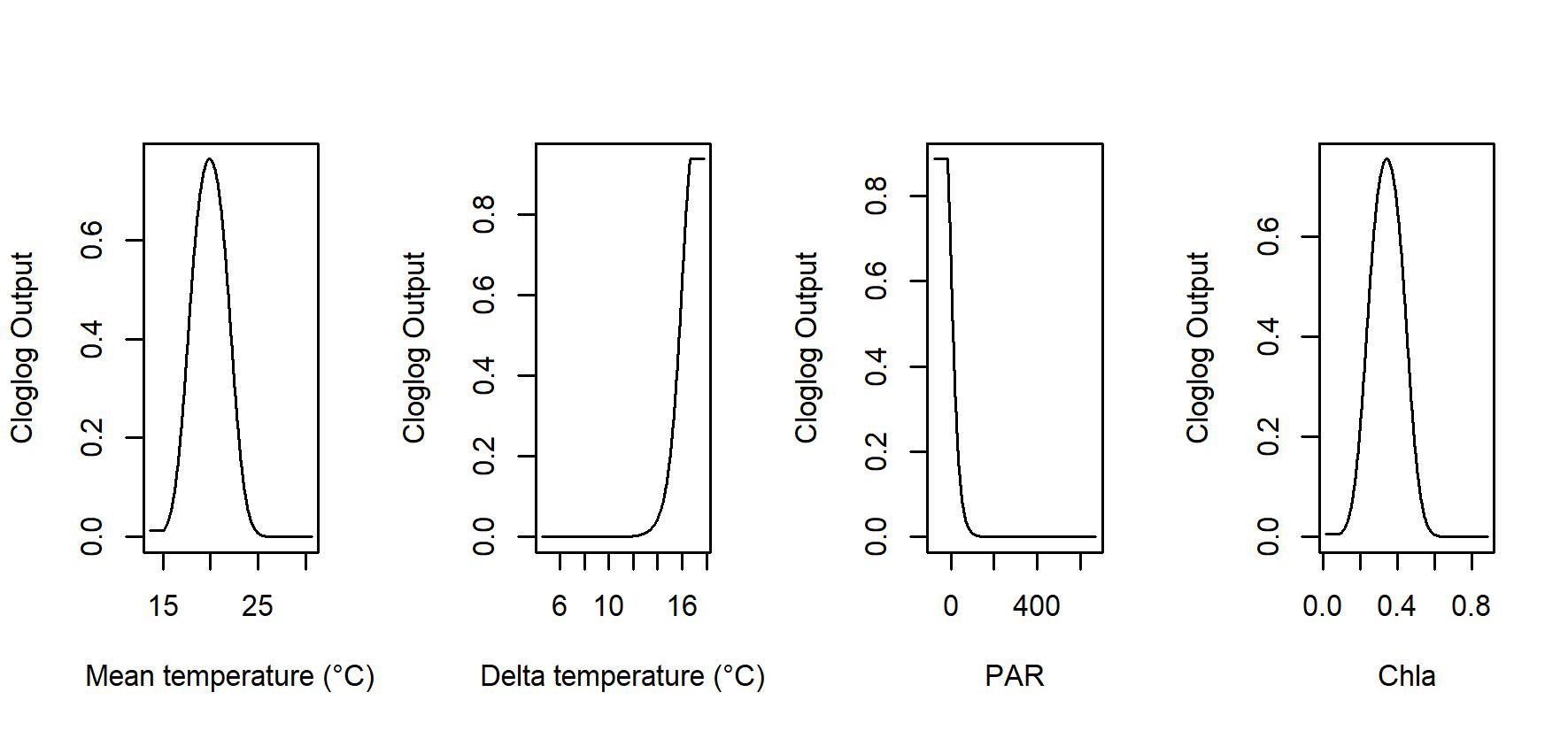


Figure S.12: Response curves after variable selection, cluster 5.

### S.3.6 Cluster 6


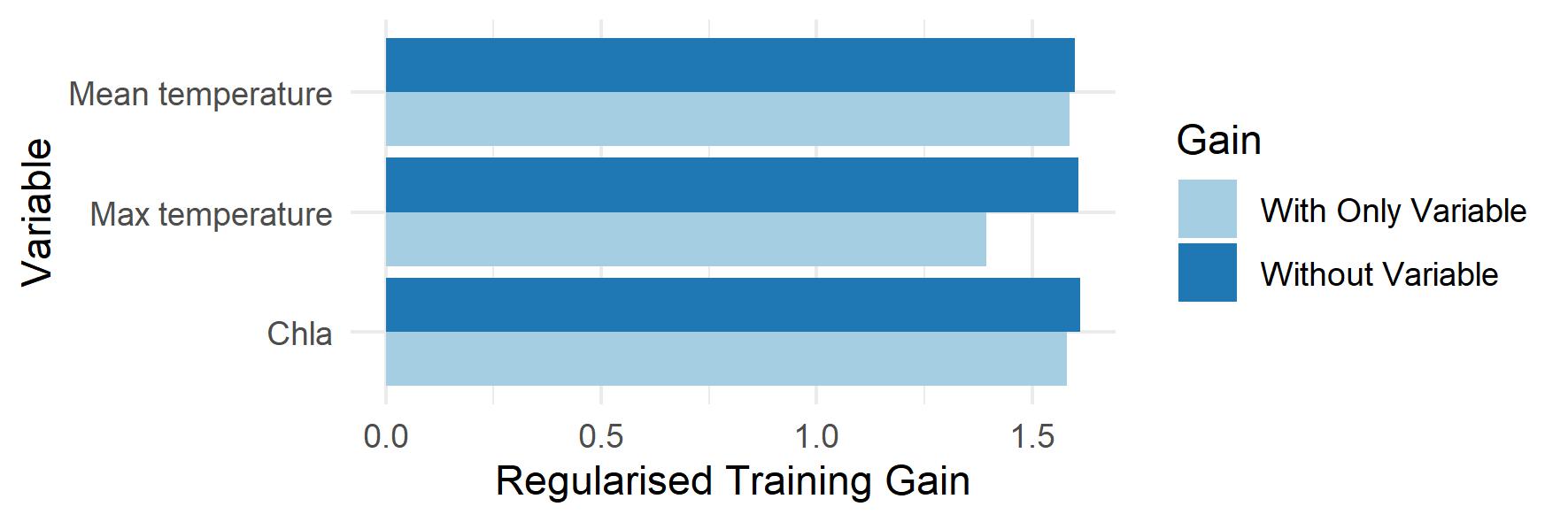


Figure S.13 : Jackknife plot of the selected variables, cluster 6.


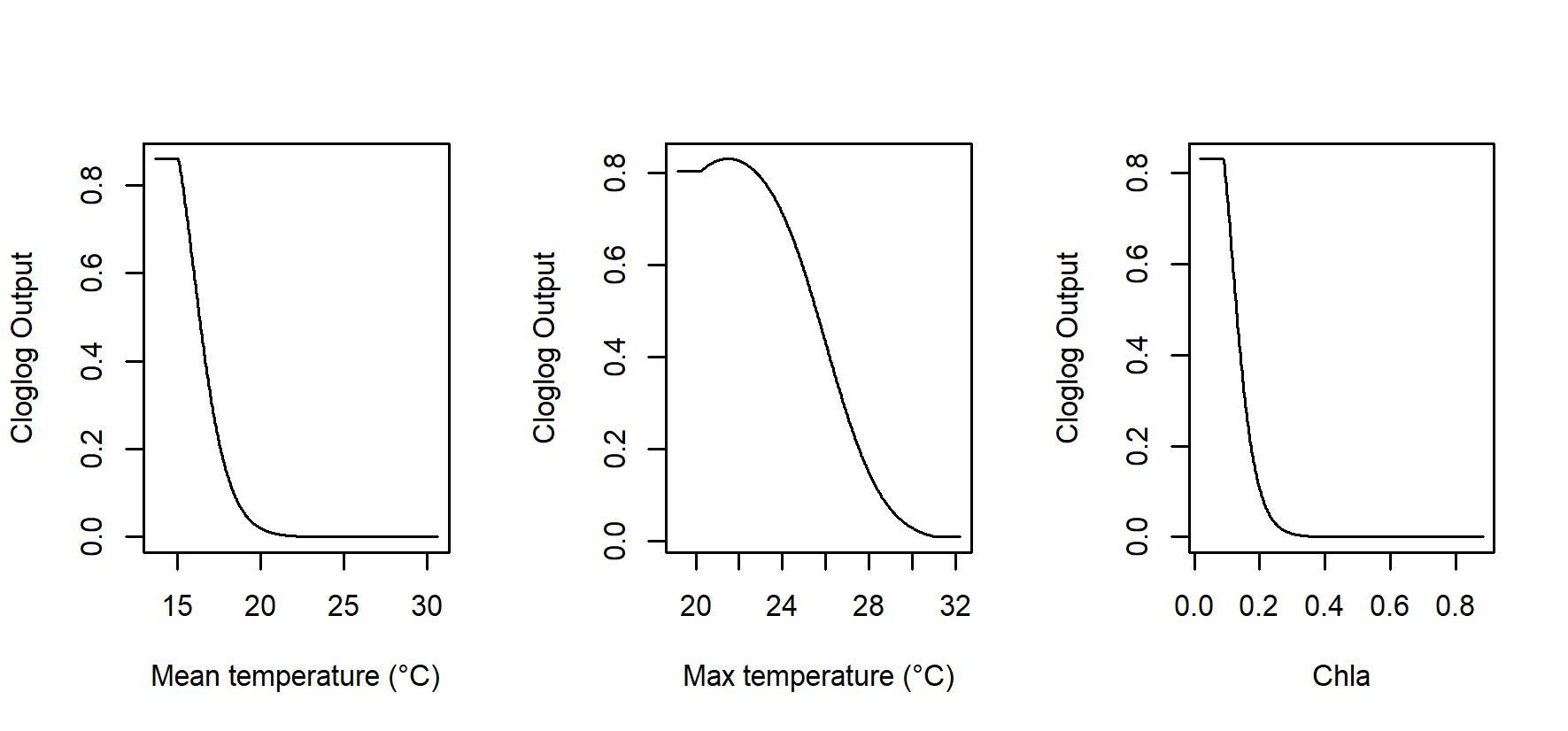


Figure S.14: Response curves after variable selection, cluster 6.

### S.3.7 MCEs – high-resolution (30-160 m)

Figure S.15 : Jackknife plot of the selected variables, cluster MCE high-resolution.


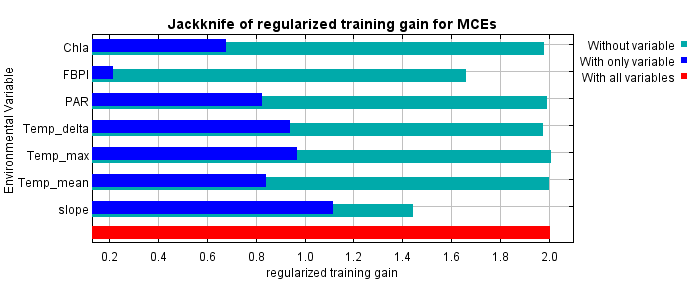


Chla

FBPI

PAR

Delta temperature

Max temperature

Mean temperature

Slope

Variable

Regularised Training Gain


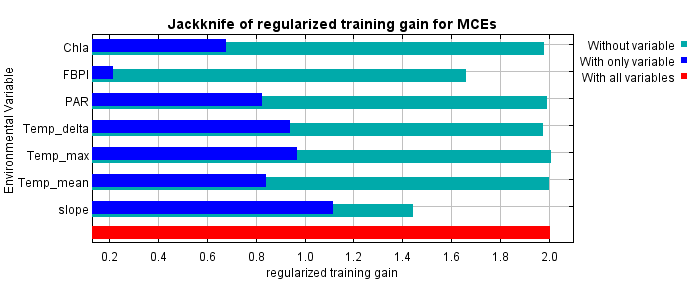


Gain

Figure S.16: Response curves after variable selection, cluster MCE high-resolution.


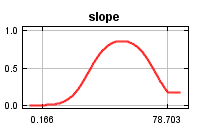

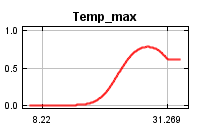

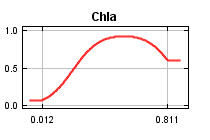

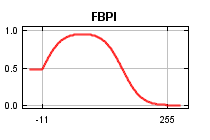

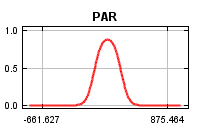

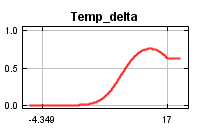


**Max temperature (°C) Mean temperature (°C)**

**Delta temperature (°C)**

**Cloglog Output**


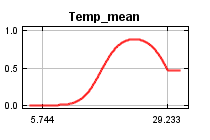


### S.3.8 MCEs – low-resolution (30-160 m)


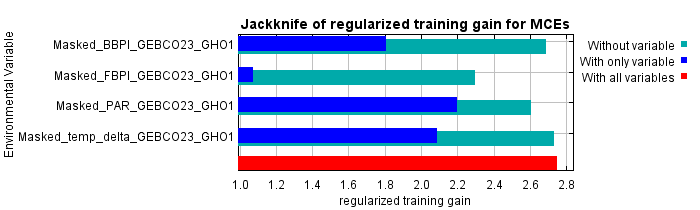


Variable

Regularised Training Gain

BBPI

FBPI

PAR

Delta temperature

Gain


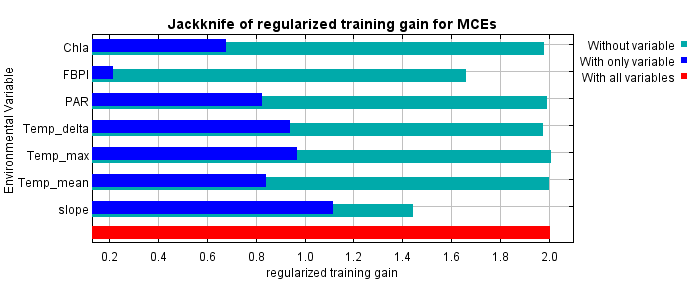


Figure S.17: Jackknife plot of the selected variables, cluster MCE low-resolution.


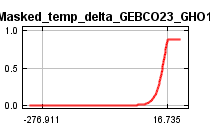


Figure S.18 : Response curves after variable selection, cluster MCE low-resolution.


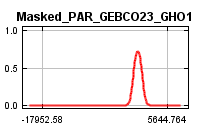


**BBPI FBPI**


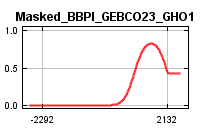

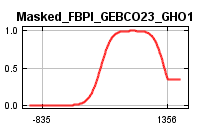


**PAR Delta temperature (°C)**

**Cloglog Output**

## S.4 Model performance results

Table S.7: Threshold-dependent evaluation indices for training, test and full models. Values given are means followed by standard deviation given in brackets. Threshold values are predicted probabilities of presence. PCC: percent correctly classified; Sens: sensitivity; Spec: specificity; thresh: threshold; Sens=Spec: sensitivity-specificity equality; MinROCdist: minimum distance to the top left corner in the ROC curve plot; MCEs high/low: MCEs cluster from high/low resolution bathymetry data. numbers are shown with 2 d.p.

| **Cluster / thresh approach** | **Average test** | | | | | **Average training** | | | | | | | **Full model** | | | | | **Thresh** | |
| --- | --- | --- | --- | --- | --- | --- | --- | --- | --- | --- | --- | --- | --- | --- | --- | --- | --- | --- | --- |
|  | PCC | Sens | | | Spec | PCC | | | Sens | Spec | | | PCC | Sens | | | Spec |  | |
| **Cluster 1** | | | | | | | | | | | | | | | | | | | |
| Sens=Spec | 0.91 (0.02) | 0.73 (0.02) | | | 0.92 (0.02) | 0.98 (0.01) | | | 0.97 (0.03) | 0.98 (0.01) | | | 0.98 (0.01) | 0.97 (0.03) | | | 0.98 (0.01) | 0.31 | |
| MinROCdist | 0.93 (0.02) | 0.73 (0.02) | | | 0.95 (0.01) | 0.98 (0.01) | | | 0.98 (0.02) | 0.98 (0.01) | | | 0.98 (0.01) | 0.97 (0.03) | | | 0.98 (0.01) | 0.31 | |
| **Cluster 2** | | | | | | | | | | | | | | | | | | | |
| Sens=Spec | 0.999  (0.001) | 1 (0) | | | 0.999  (0.001) | 0.995  (0.004) | | | 1 (0) | 0.99 (0.004) | | | 0.995 (0.003) | 1 (0) | | | 0.99 (0.004) | 0.31 | |
| MinROCdist | 0.999  (0.001) | 1 (0) | | | 0.999  (0.001) | 0.995  (0.004) | | | 1 (0) | 0.99 (0.004) | | | 0.995 (0.003) | 1 (0) | | | 0.99 (0.004) | 0.31 | |
| **Cluster 3** | | | | | | | | | | | | | | | | | | | |
| Sens=Spec | 0.889  (0.018) | 0.838  (0.039) | | | 0.902 (0.028) | 0.990 (0.007) | | | 0.9841 (0.016) | 0.985 (0.008) | | | 0.986  (0.006) | 0.989 (0.011) | | | 0.985 (0.007) | 0.335 | |
| MinROCdist | 0.894 (0.017) | 0.854 (0.030) | | | 0.904 (0.022) | 0.990 (0.006) | | | 0.998  (0.002) | 0.987 (0.007) | | | 0.988 (0.005) | 1 (0) | | | 0.985 (0.007) | 0.225 | |
| **Cluster 4** | | | | | | | | | | | | | | | | | | | |
| Sens=Spec | 0.990  (0.007) | 1 (0) | | | 0.988  (0.009) | 0.987  (0.008) | | | 0.992  (0.008) | 0.986 (0.009) | | | 0.986  (0.006) | 0.986  (0.014) | | | 0.986  (0.006) | 0.21 | |
| MinROCdist | 0.990  (0.007) | 1 (0) | | | 0.988  (0.009) | 0.987  (0.008) | | | 1 (0) | 0.985  (0.009) | | | 0.988  (0.005) | 1 (0) | | | 0.986  (0.006) | 0.19 | |
| **Cluster 5** | | | | | | | | | | | | | | | | | | | |
| Sens=Spec | 1 (0) | 1 (0) | | | 1 (0) | 1 (0) | | | 1 (0) | 1 (0) | | | 1 (0) | 1 (0) | | | 1 (0) | 0.37 | |
| MinROCdist | 1 (0) | 1 (0) | | | 1 (0) | 1 (0) | | | 1 (0) | 1 (0) | | | 1 (0) | 1 (0) | | | 1 (0) | 0.37 | |
| **Cluster 6** | | | | | | | | | | | | | | | | | | | |
| Sens=Spec | 0.970 (0.01) | | 0.970 (0.03) | 0.970  (0.016) | | | 0.960  (0.011) | 0.965  0.027 | | | 0.959  (0.013) | 0.962  (0.009) | | | 0.955  (0.026) | 0.963  (0.010) | | | 0.455 |
| MinROCdist | 0.975  (0.013) | | 1 (0) | 0.970  (0.016) | | | 0.964  (0.011) | 1 (0) | | | 0.957 (0.013) | 0.967 (0.009) | | | 1 (0) | 0.960  (0.010) | | | 0.24 |
| **MCEs – high-resolution** | | | | | | | | | | | | | | | | | | | |
| Sens=Spec | 0.930  (0.003) | | 0.932  (0.047) | 0.930 (0.003) | | | 0.930  (0.003) | 0.930  (0.014) | | | 0.930 (0.003) | 0.930  (0.003) | | | 0.929  (0.013) | 0.930 (0.003) | | | 0.31 |
| MinROCdist | 0.924  (0.003) | | 0.963  (0.034) | 0.924 (0.003) | | | 0.921  (0.003) | 0.958  (0.011) | | | 0.920 (0.003) | 0.927  (0.003) | | | 0.948  (0.011) | 0.927 (0.003) | | | 0.29 |
| **MCEs – low-resolution** | | | | | | | | | | | | | | | | | | | |
| Sens=Spec | 0.992  (0.001) | | 1 (NA) | 0.992 (0.001) | | | 0.989  (0.001) | 0.933 (0.041) | | | 0.989 (0.001) | 0.990 (0.001) | | | 1 (0) | 0.990 (0.001) | | | 0.75 |
| MinROCdist | 0.992  (0.001) | | 1 (NA) | 0.992 (0.001) | | | 0.989  (0.001) | 0.933 (0.041) | | | 0.989 (0.001) | 0.990 (0.001) | | | 1 (0) | 0.990 (0.001) | | | 0.75 |

##
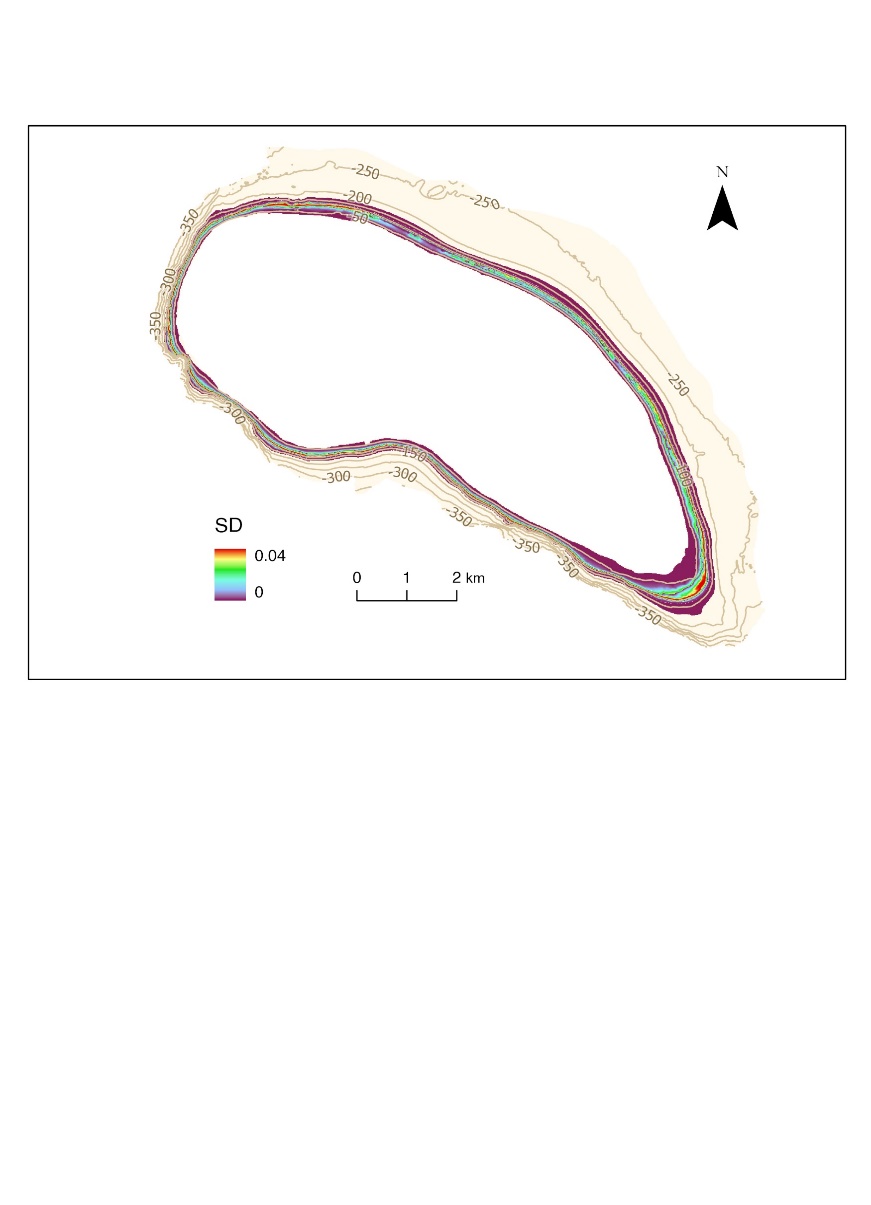
S.5 Predicted standard deviation maps

Figure S.19 : Predicted standard deviation maps for each cluster assemblage at Egmont Atoll, using MaxEnt modelling. With depth contours at 50 m intervals, starting at 50 m deep. a. Cluster 1; b. Cluster 2; S. Cluster 3; d. Cluster 4; e. Cluster 5; f. Cluster 6.

**d**


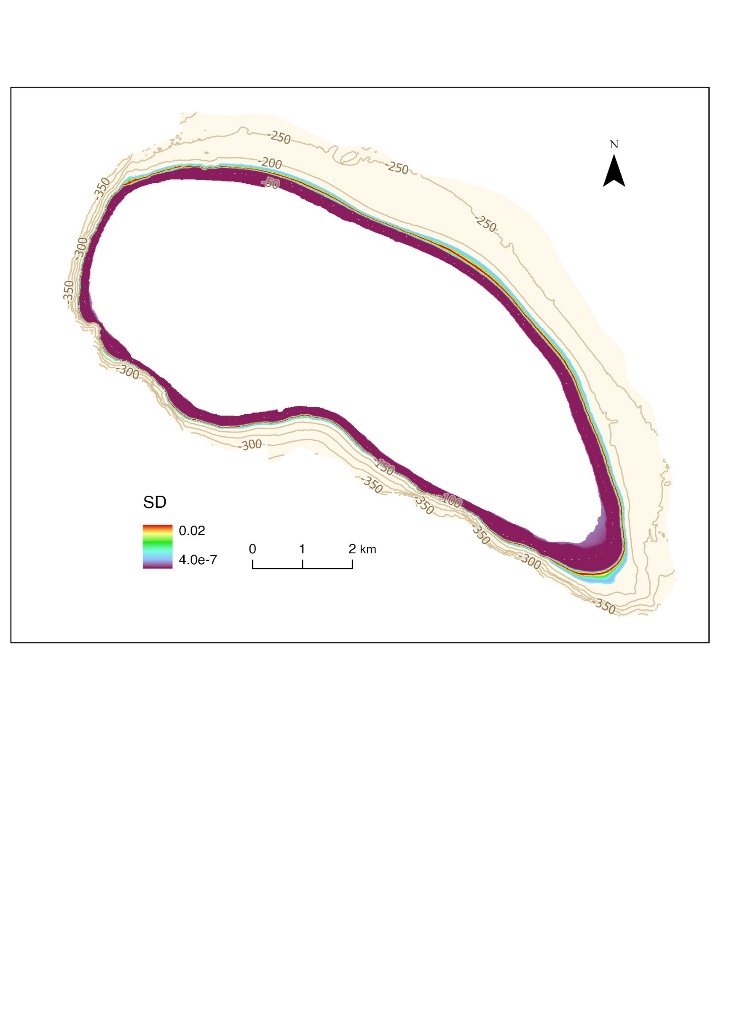

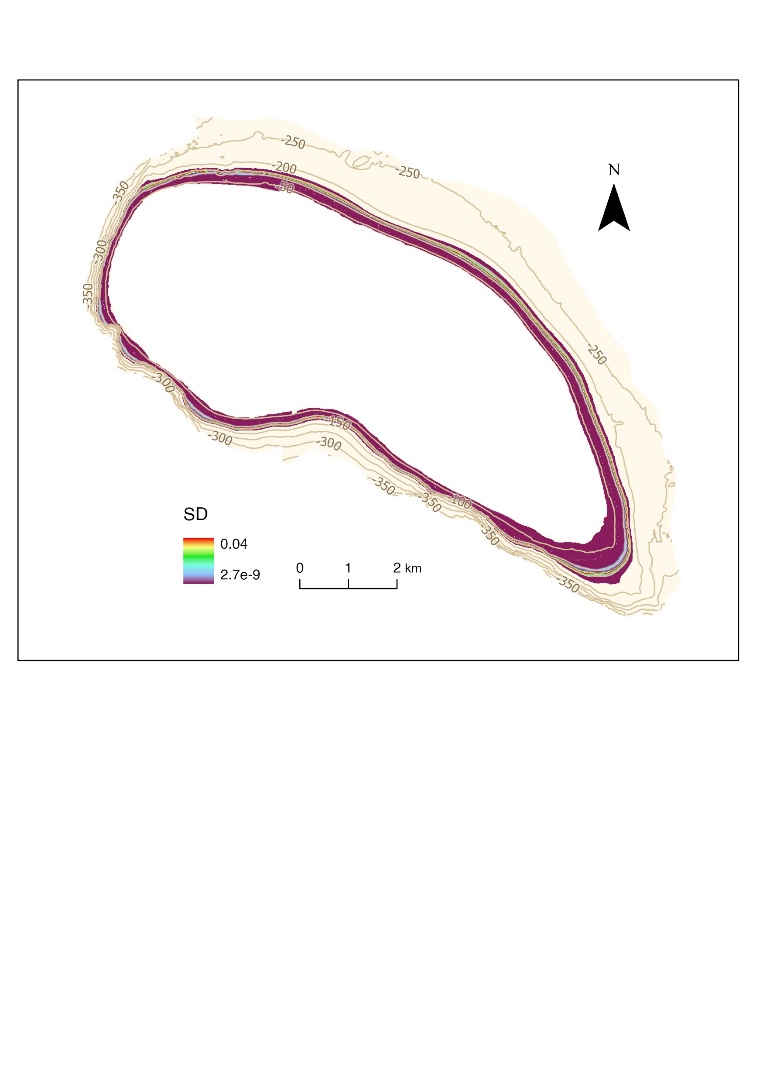

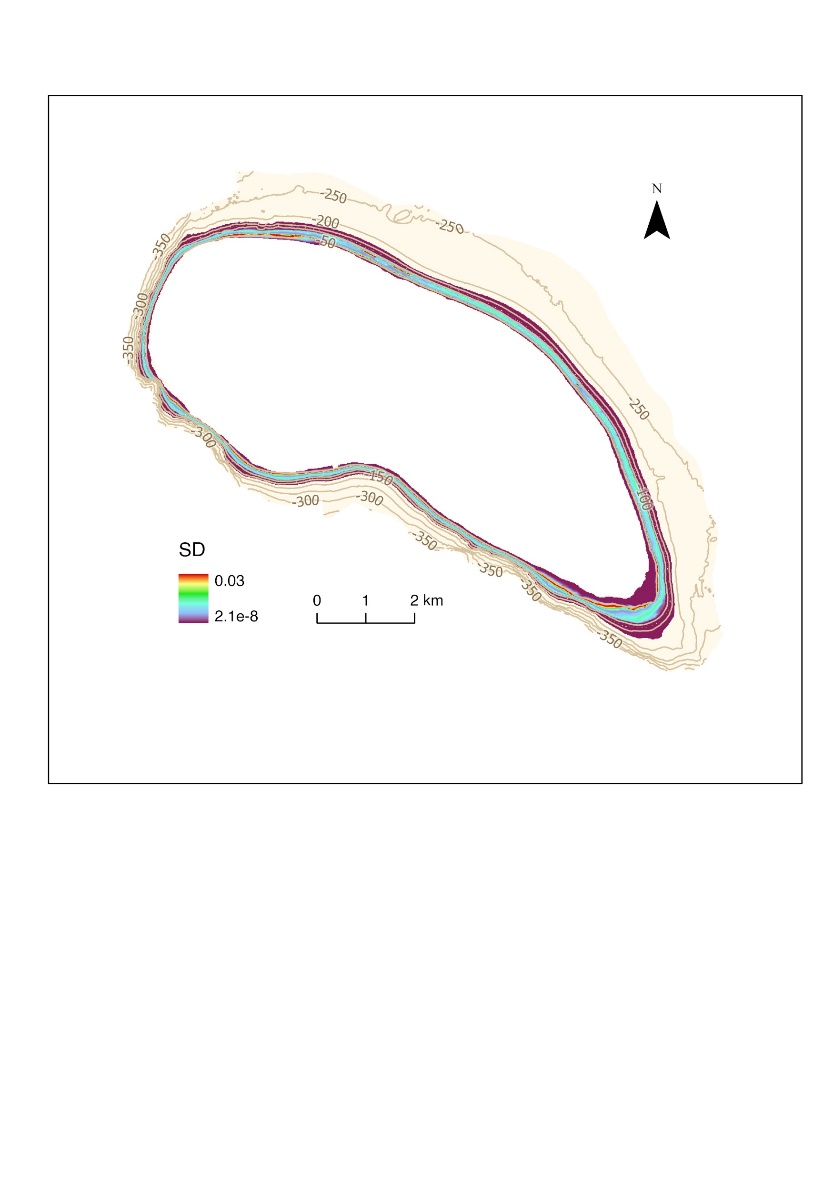

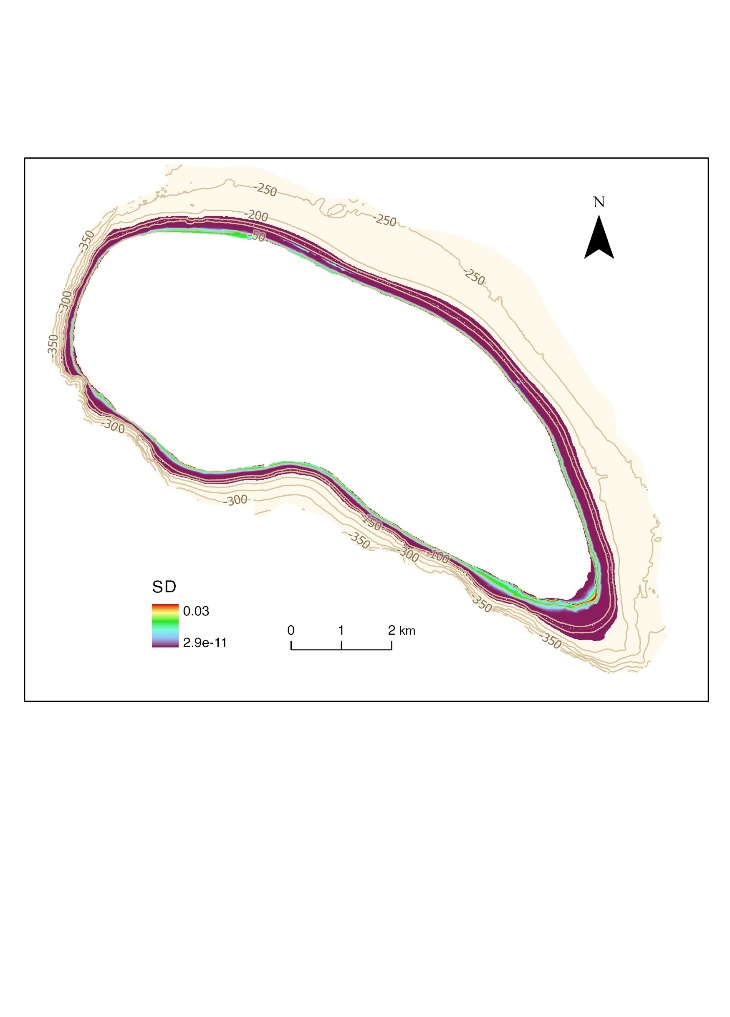

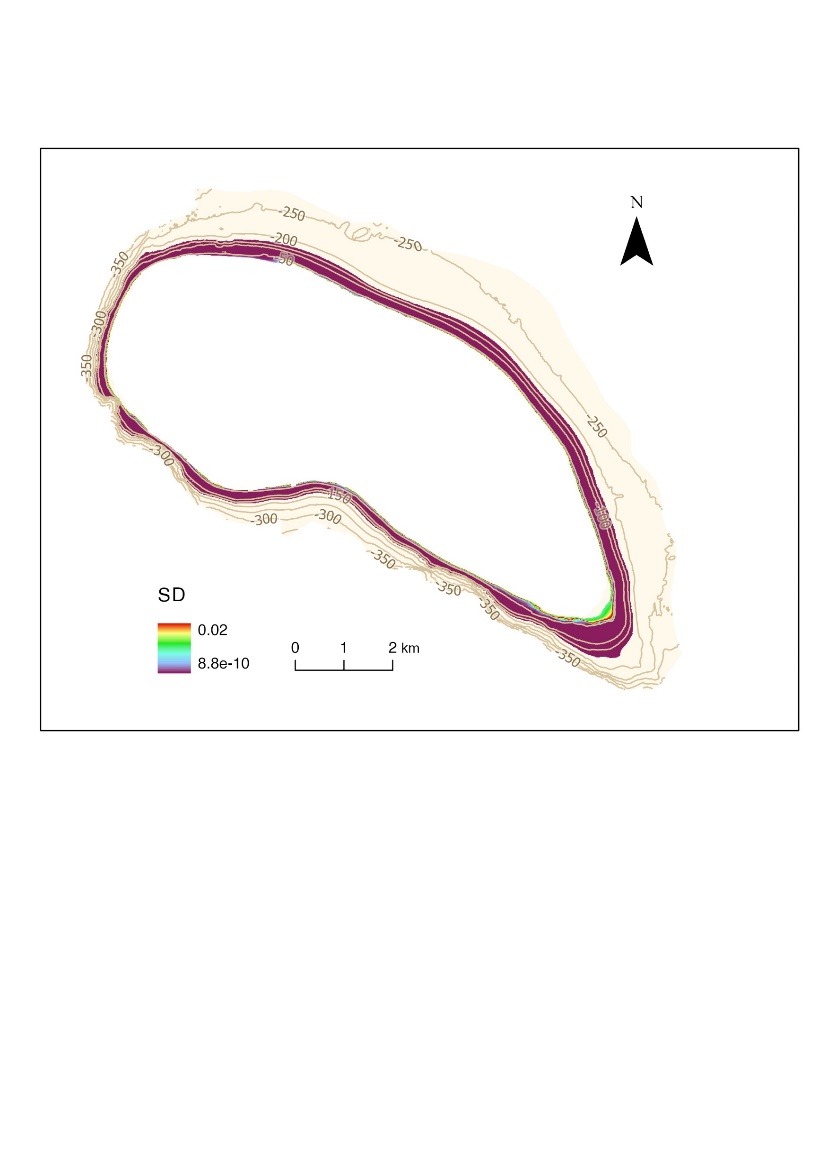


**a**

**b**

**e**

**c**

**f**


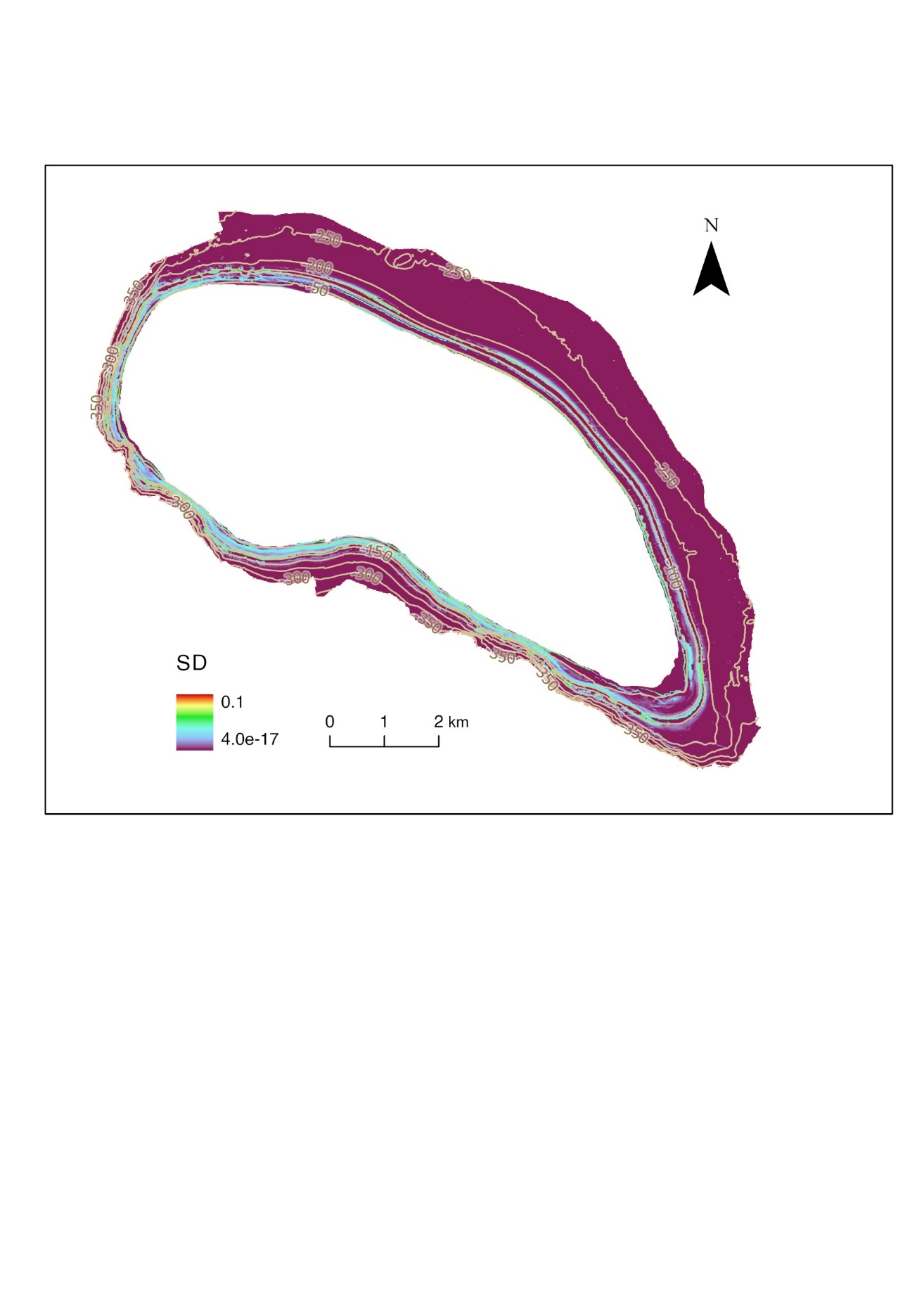


Figure S.20: Predicted standard deviation maps for MCEs – high-resolution bathymetry data cluster at Egmont Atoll, using MaxEnt modelling. With depth contours at 50 m intervals, starting at 50 m deep.

Figure S.21 : Predicted standard deviation maps for MCEs – low-resolution bathymetry data cluster of the Chagos Archipelago, using MaxEnt modelling. With depth contours at 50 m intervals, starting at 50 m deep to 200 m, then showing the 1000 m depth contour.


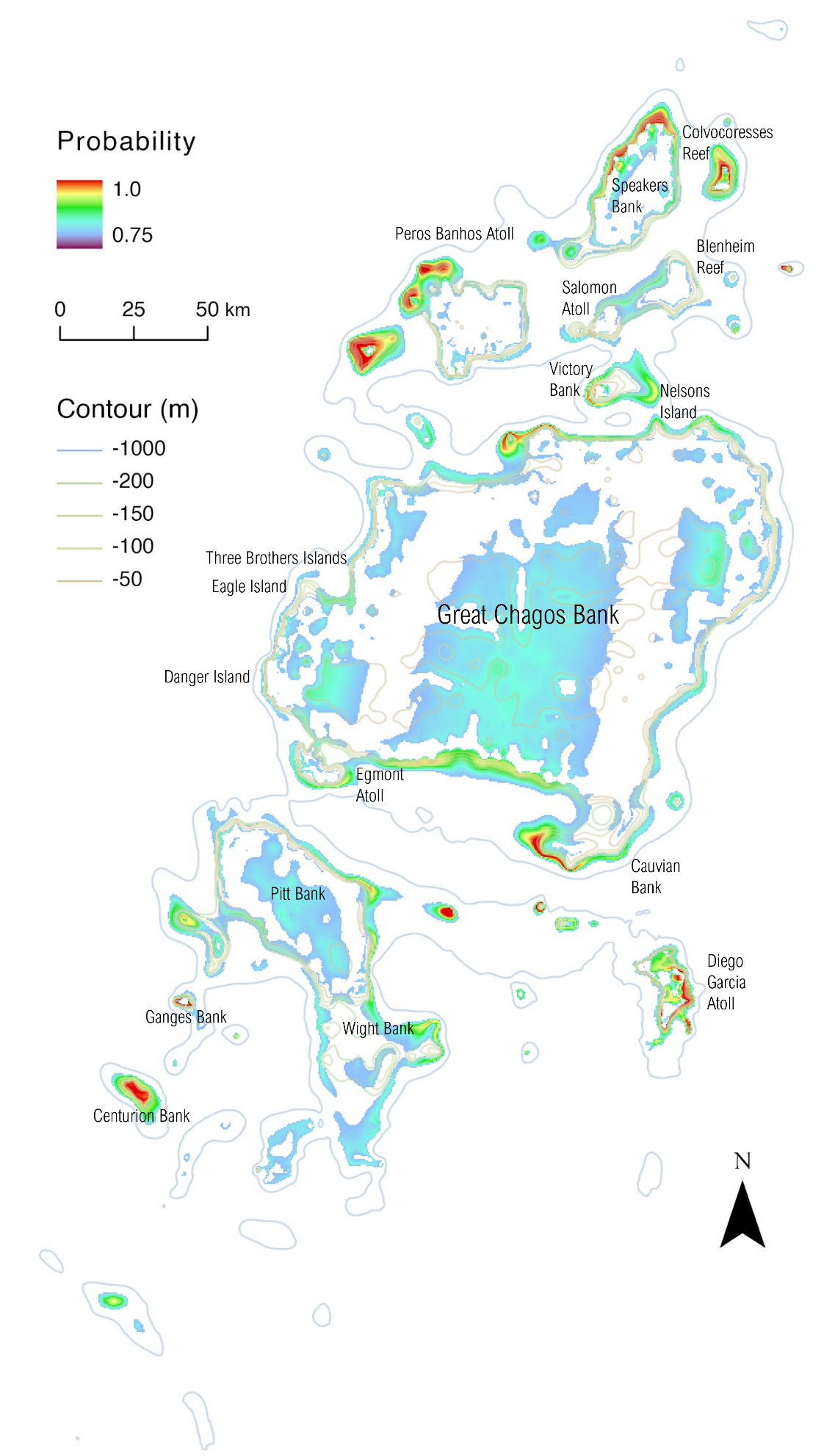

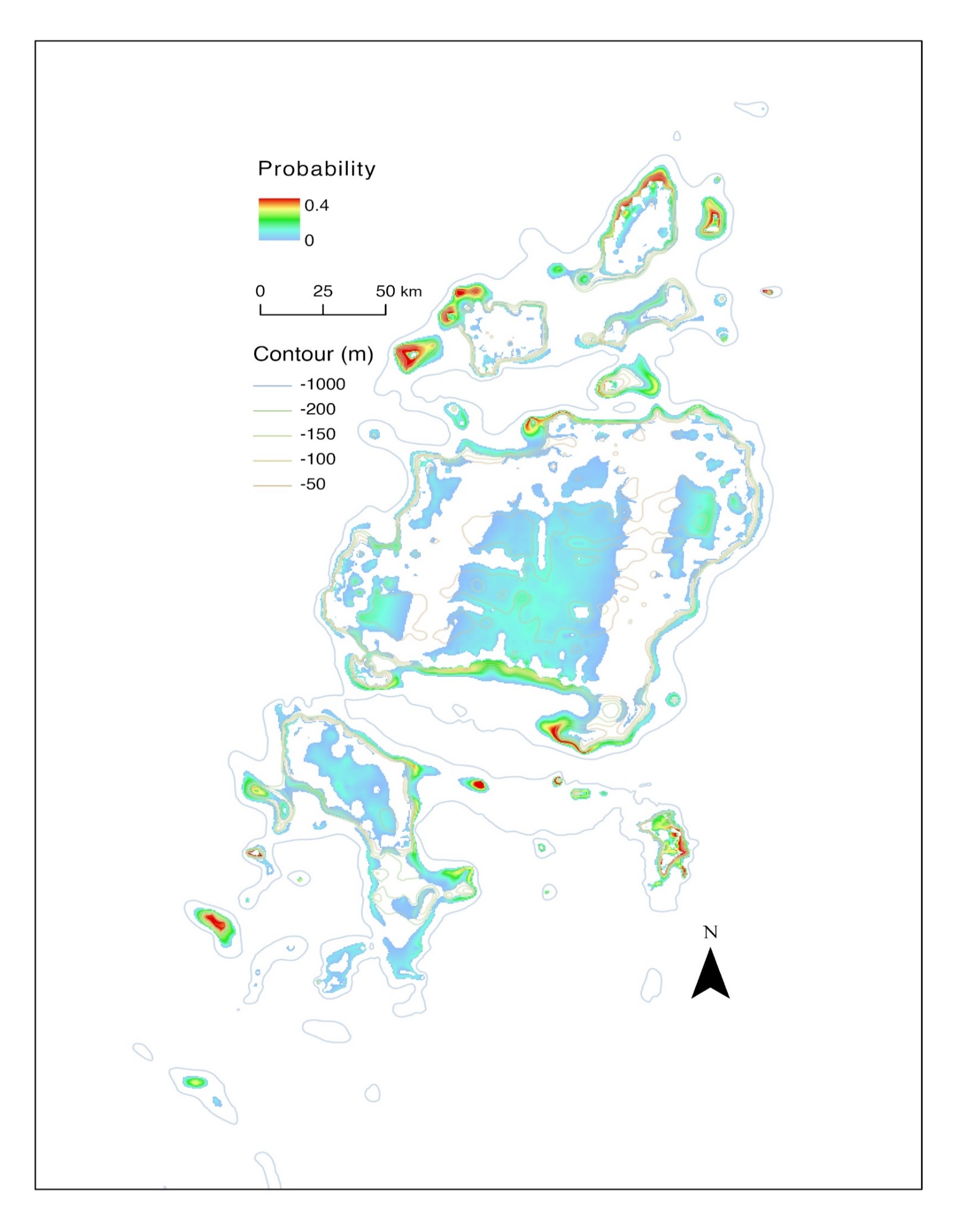


##

## S.6 Egmont multibeam – GEBCO comparison


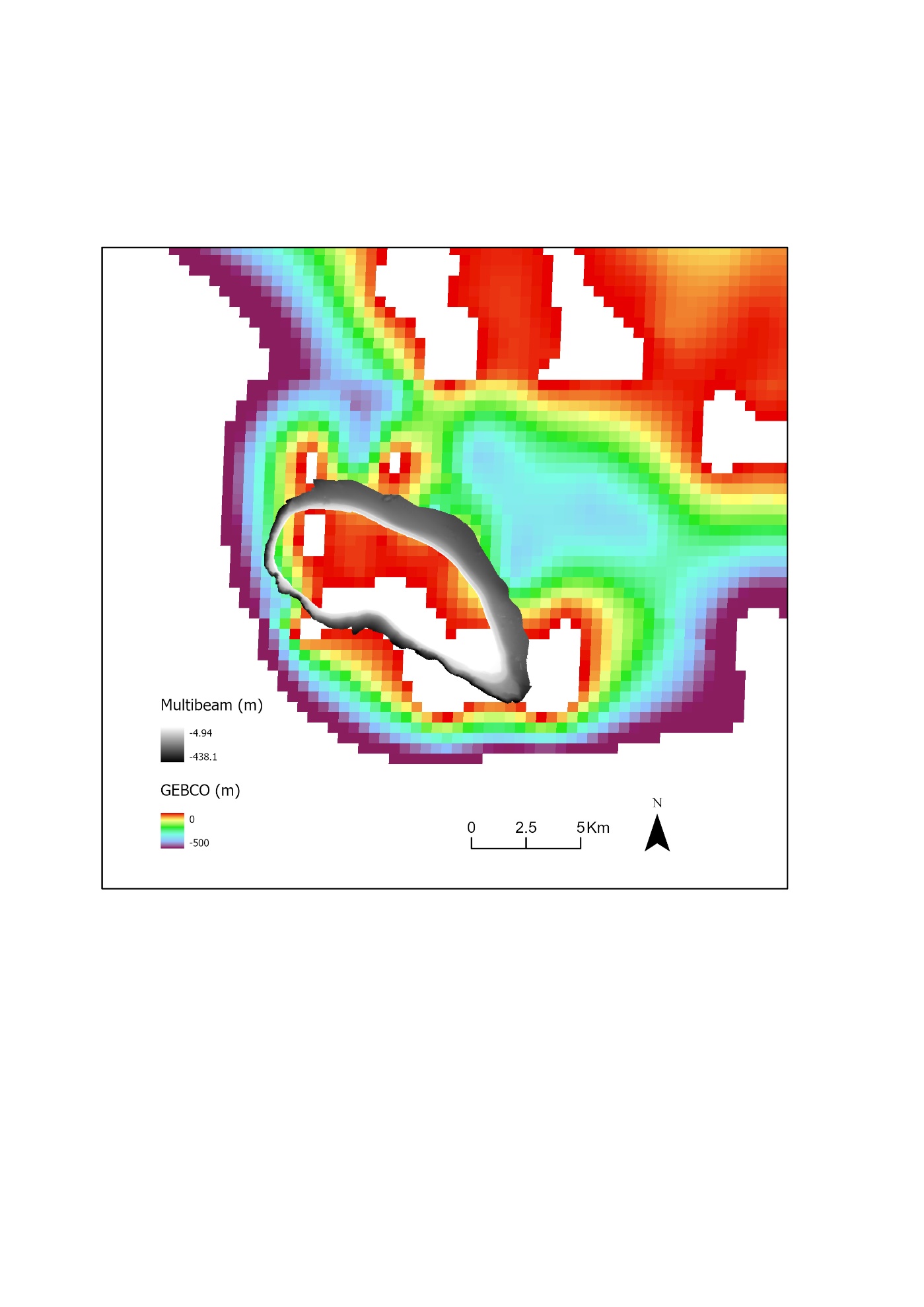


Figure S.22: Overlapped bathymetry data (m) in Egmont Atoll, from multibeam (grey scale) and GEBCO (rainbow scale) models.
